# Root and canal anatomy of mandibular first molars using micro-computed tomography: a systematic review

**DOI:** 10.1186/s12903-023-03036-5

**Published:** 2023-05-29

**Authors:** Hasan M. AL-Rammahi, Wen Lin Chai, Mohamed Shady Nabhan, Hany M. A. Ahmed

**Affiliations:** 1 Department of Restorative Dentistry, Faculty of Dentistry, University of Malaya, 50603 Kuala Lumpur, Malaysia; 2 Department of Conservative Dentistry, Faculty of Dentistry, University of Babylon, AL Hillah city, Iraq; 3 Department of Prosthetic Dentistry, Faculty of Dentistry, Ain Shams University, Cairo, Egypt

**Keywords:** Mandibular first molar, Micro-CT, Root and canal anatomy, Review

## Abstract

**Background:**

A thorough understanding of root and canal anatomy is crucial for successful root canal treatment outcomes. This systematic review aims to explore the published micro-CT studies investigated the anatomy of root and canal system in permanent mandibular first molars.

**Method:**

An electronic search was performed on Web of science, PubMed, and Scopus. Micro-CT journal studies investigated the root and canal anatomy of permanent double-rooted mandibular first molars were included. Data on study characteristics, objectives of interest, specifications of the studies, and micro-CT specifications were extracted. Risk of bias assessment (ROB) of the included studies was performed using Anatomical Quality Assessment (AQUA) tool. The extracted data were presented in tables and figures to present and synthesise the results. A meta-analysis was performed for the studies related to the prevalence of Vertucci's canal configurations, middle mesial canal (MMC) configurations, and Fan's isthmus types.

**Results:**

Amongst 1358 identified studies, thirty met the inclusion criteria. In terms of the objectives, the selected studies showed high anatomical variability in mandibular first molars. Twenty-two (73%), 25 (83%), and 12 (40%) of the studies reported the population/ethnicity, micro-CT specifications, and ethical approval, respectively. 28 (93%) studies did not disclose the method of sample size estimation. In only 6 (20%) of the studies, the authors had calibrated the assessment approaches. Mostly, a potential ROB was reported in domain 1 (objective(s) and subject characteristics) and domain 3 (methodology characterization). Whilst, low risk was reported in domains 2 (study design), 4 (descriptive anatomy), and 5 (reporting of results). The overall ROB was reported to be ''moderate'' in the vast majority of the studies (27/30). Meta-analysis results showed high levels of heterogeneity among the studies related to MMCs (I^**2**^ = 86%) and Fan's isthmus (I^**2**^ = 87%). As for the root canal configuration, pooled prevalence showed that Vertucci type IV and type I were the most prevalent in mesial and distal root canals, respectively.

**Conclusion:**

Based on moderate risk of bias level of evidence, micro-CT studies have shown wide range of qualitative and quantitative data presentations of the roots and canals in mandibular first molars.

Protocol and registration.

The protocol of this systematic review was prospectively registered in the Open Science Framework database (https://osf.io) on 2022–06-20 with the registration number 10.17605/OSF.IO/EZP7K.

## Introduction

In-depth awareness of the root and root canal system morphology is crucial to ensure the success of non-surgical and surgical root canal procedures [1, 2]. To reduce or prevent the potential mishaps and failures in root canal treatment, endodontists must have a comprehensive awareness of the anatomical challenges in the root canal system [3].

The internal and external root and canal morphology of the mesial [4] and distal roots [5] were reported to be highly variable in the mandibular first molar. Ethnicity, age, gender, and study design are the most apparent factors that can contribute to that high variability [6, 7]. Such anatomical variations may complicate the mission of achieving successful root canal treatment. Amongst all human teeth, the mandibular first molar is often affected by caries and usually requires root canal treatment, even at an early age [8]. Variation in the number of roots, which ranges from one [9] to four [10], and in the morphology of the root, such as proximal grooves [11], are common anatomical variations in mandibular first molars. The occurrence of a distolingual (radix entomolaris) or mesiobuccal (radix paramolaris) supernumerary root was not uncommon in this tooth type and found to be closely associated with many ethnicities, such as Mongoloid, Brazilian, and Chinese [12–14]. Failure to localise and treat the extra root's canal can lead to persistent canal infection and, hence, failed root canal treatment [15].

Investigations have shown that mandibular first molars possess three or four canals [15, 16], which are characterised by high complexity due to anatomical variations such as isthmuses, fins, accessory canals, splitting and merging canals at different levels of the roots as well as. For instance, the existence of isthmuses and intercanal communications in the mesial root provides enough room for the accumulation of hard tissue debris during canal instrumentation [17]. These difficult-to-reach areas could require more advanced irrigation materials and techniques to improve the removal of the impacted debris [18]. It has also been reported that chemicomechanical cleaning of isthmus-containing canals require the use of adjunctive steps to enhance the treatment outcome [17, 19, 20]. For instance, XP-endo (Finisher R) significantly enhanced removal of remnant filling material from the isthmus containing canals retreated with Mtwo instrument system (VDW) [20]. Ultrasonic activation resulted in the highest mean debris reduction in isthmuses compared to the conventional techniques [17]. Curved canals could also pose a substantial difficulty for practitioners due to the increased likelihood of procedural mishaps such as apical transportation, ledge formation, and perforation [21].

Previous works have shown the existence of an extra mesial root canal between the mesiobuccal and mesiolingual canals, named as the middle mesial canal [22–24], with a reported prevalence of up to 46% [25]. The middle mesial canals (MMCs) have been characterised by having a deep, small entrance mostly within the isthmus or a developmental groove between the mesiobuccal and mesiolingual root canal orifices [26]. It has been noted that MMCs are difficult to detect and access [26]. Inability to identify a MMC might cause treatment failure, as effective root canal treatment relies on complete chemomechnical cleaning of the root canal system [26].

The presence of thin dentine on the furcal aspect of the mesial root for a considerable apical distance and, subsequently, a narrow mesiodistal dimension, could have serious clinical implications. On the mandibular first molar, a 51.8% incidence of vertical root fracture (VRF) was observed [27]. Regarding endodontically treated teeth, the occurrence of VRF in mandibular first molars is almost twice that in maxillary first molars. Fractures also took place more frequently in flat, thin roots with narrower mesio-distal dimensions, like the mesial roots of mandibular molars [28].

Numerous research methods have been used to study the root canal morphology of the permanent mandibular first molars. They include the resin injection method [29], two-dimensional (2D) radiographic imaging with and without hand files inserted into root canals [30], a clearing technique with the use of a dye [31, 32], stereomicroscopy [33], scanning electron microscopy (SEM) [34], computed tomography (CT) [33], cone-beam computed tomography (CBCT) [35], and micro-computed tomography (micro-CT) [36]. According to a recent review, attention has been paid to the application of micro-CT in different research areas in endodontics, such as root canal preparation (23.8%), canal system anatomy (17.4%), canal obturation (9.2%), and retreatment (7.0%) of the root canal system [37].

Over the last two decades, micro-CT technology has been increasingly used to investigate a wide range of internal and/or external root and canal anatomical features in different tooth types. It has been evidently reported that micro-CT is referred to be as, compared to CBCT and other earlier evaluation modalities, the "gold standard" in investigating the canal length [38], cross-sectional shape [39], canal configuration [40, 41], apical deltas and number of foramen [42], canal curvature [43], isthmus [44], and intercanal communication and accessory canals [45]. Its non-destructive nature and reproducibility, smaller voxel size, higher spatial resolution, higher degree of rotation (360°), and thinner slice thickness contribute to its powerful ability to accurately identify extremely fine and highly complicated anatomies. However, the drawbacks of this technology are its expensive cost, prolonged imaging time, requiring high data storage, and the fact that its usefulness is confined to laboratory study models (mainly for research purposes) and, up to date, it cannot be used in clinical scenarios. Micro-CT also requires well-trained operators with different image processing software programs, in addition to the high cost of the software licenses.

Systematic reviews have become increasingly important to identify, analyse, and evaluate root and canal anatomy studies [4, 23, 46]. Owing to the considerable data presentations along previous decade, this systematic review aimed to discuss the internal and external anatomical details of mesial and distal roots in the permanent mandibular first molar using micro-CT.

## Methodology

This systematic review followed the Preferred Reporting Items for Systematic Reviews and Meta-Analysis (PRISMA 2020) statement guidelines.

### Search query and Information sources

**Fig. 1:**
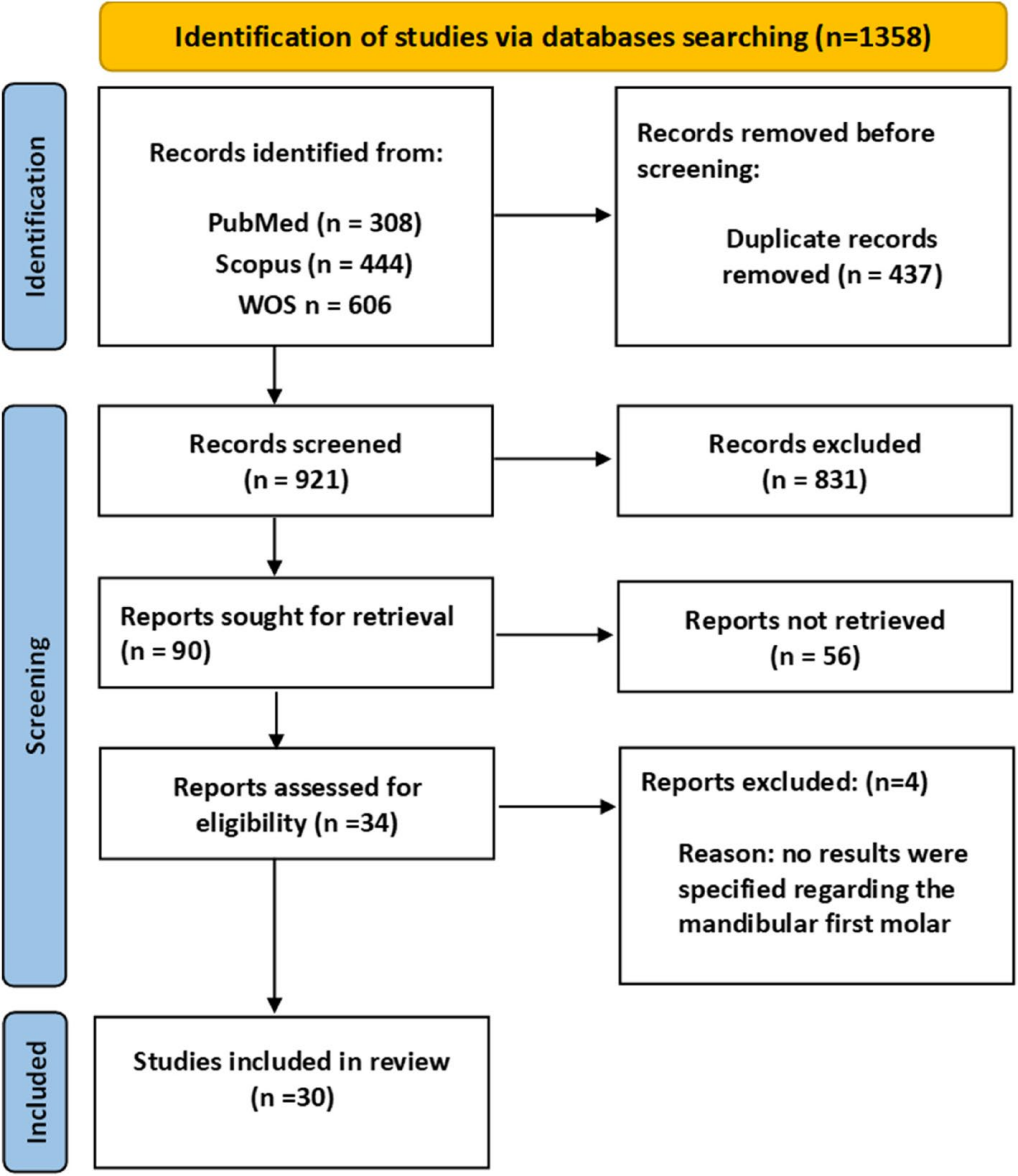
PRISMA Flowchart of study search

Figure 1 (PRISMA Flowchart of Study Search) shows the approach for the literature search and the search results. A literature search was undertaken in three search engines – Web of Science, PubMed, and Scopus – to identify articles related to micro-CT evaluation of the root and root canal anatomy of mandibular first molars. Given that the field of interest is micro-CT-based anatomy of the mandibular first molar, ‘Mandibular first molar’' OR ‘Mandibular 1^st^ molar’ OR ‘Mandibular molar’ OR ‘Mandibular molars’ AND ‘Micro-CT’ OR ‘Micro CT’ OR ‘MicroCT’ OR ‘Microcomputed tomography’ OR ‘Micro-computed tomography’ were used as keywords. The rationale behind using different spellings of the search query words is that these words have been used interchangeably in the literature. The settings of the search query on the relevant databases are shown in Table 1.

**Table 1:** Specifications of search query

**Database**	**PubMed**	**Scopus**	**Web of Science**
**Search period**	Jan 2000 – Oct 2021	Jan 2000 – Oct 2021	Jan 2000 – Oct 2021
**Study language**	English	English	English
**Search in**	Title/Abstract	Titles, abstract & keywords	Topic
**Date of conducting the search query**	October 2021	October 2021	October 2021

### Inclusion and exclusion criteria

#### Anatomy-based criteria

Micro-CT investigations of permanent human tooth root and/or root canal morphological variations in the mandibular first molar such as the number of roots, quantitative and/or qualitative evaluation of the danger zone (thinnest root dentine), the configuration of root canals, canal isthmuses, major and/ or minor apical foramen (apical constriction) morphology, and accessory canals were included. Root or canal anomalies such as *radix entomolaris*, *radix paramolaris*, and taurodontism were excluded.

#### Publication type–based criteria

In this systematic review, experimental micro-CT-based studies were included. Case reports on extracted teeth were excluded. Conference papers, pilot studies, editorials, and reviews were excluded. Articles published in languages other than English were excluded.

#### Purpose-based criteria

All studies related to root and canal anatomy that compared different diagnostic tools and examined one method other than micro-CT (such as clearing and staining and CBCT) were excluded. No restraints regarding the number of scanned teeth were established, as the sample size issue was considered a criterion during the ROB assessment.

#### Tooth type-based criteria 

Articles that investigated the primary molars or permanent teeth other than the mandibular first molar were excluded. Studies investigating both first and second mandibular molars (or mandibular molars in general) but did not mention specific results for each molar type separately were excluded. Studies investigating three-rooted mandibular first molars were excluded as well.

### Evaluation of the selected studies

Four rounds of screening and filtering were performed in the article selection process. First, duplicates were deleted. Second, unrelated articles were screened out through titles. Third, unrelated articles were screened out through abstracts. Finally, full texts of related articles were read and analysed. Two independent authors assessed the inclusion and exclusion of the articles based on the established criteria. There was an almost perfect interobserver agreement based on Cohen's kappa coefficient (kappa = 0.88). In the case of disagreement regarding the inclusion and exclusion of a retrieved article, a consensus was reached by a discussion between authors.

Tables 2 and 3 show the main characteristics of the selected studies. The following information was extracted and tabulated from each selected study: author(s), year of publication, population/ethnicity, sample size calculation, diagnostic device (micro-CT) specifications and software used, evaluation process (2D or 3D), assessment of reliability approach (inter or intra-rating), ethical approval, type of study (retrospective or prospective), type of analysis (qualitative and/or quantitative), and classification used. The objectives of interest of each selected study were extracted, tabulated, and ticked (**✓**) if they were included in a given study (Table 3). Additionally, using MetaXL software (version 5.3, EpiGear International, 2016), a meta-analysis was performed for the studies related to the prevalence of Vertucci's main canal configurations, middle mesial canal (MMC) configurations, and Fan's isthmus types.

**Table 2:** Characteristics of the included studies

	**Studies**	**Population/ ethnic group**	**Sample size and (its estimation method)**	**Diagnostic device specifications and technique**	**Evaluation process**	**Ethical approval**
**1.**	Mannocci et al. [47]	Not reported	20 (Not reported)	100 kV to achieve a focal spot of 6 µm. The sample was positioned between the source and the detector to achieve X4 magnification. Two-dimensional individual images with a pixel size of 12.5 µm, and a slice thickness of 25.0 µm were obtained	Axial sections of 3D images	Not reported
**2.**	Iwaka et al. [48]	Japanese	30 (Not reported)	45 kV, 100 μA and slice width, 16.5 μm. Prior to imaging, each tooth was set such that the sample stage was orthogonal to the tooth axis. (PHOTOSHOP 6.0, Adobe inc., Sanjose, CA, USA) and (VOXBLAST, Vaytec Inc., Los Angeles, CA, USA	2D & 3D	Patients' agreements
**3.**	Gu et al. [49]	Chinese	36 (Not reported)	Voxel sizes of 15 × 15 × 15 mm, and the cross-section was perpendicular to the long axis of the root	2D	Not reported
**4.**	Gu et al.[12]	Chinese	122, then 25 2-rooted were selected (Not reported)	Each specimen was scanned along the teeth axis with voxel sizes of 21 mm × 21 mm × 21 mm. The resulting data were then processed by software Microview 2.1.2 (GE HealthCare, London, Ontario, Canada)	2D &3D	Not reported
**5.**	Gu et al. [50]	Chinese	Initially 122, then 25 were selected (Not reported)	Each specimen was scanned along the teeth axis with voxel sizes of 21 × 21 × 21 mm. The data sets (DICOM format) were transferred to Mimics 10.01 (Materialise, Leuven, Belgium) software	3D	Not reported
**6.**	Fan et al. [51]	Chinese	70 (not reported)	37-mm intervals, with the scanning being perpendicular to the long axis of the teeth. (3D-Doctor; Able Software Corp, Lexington, MA) was used for 3D reconstruction and image analysis	3D	Not reported
**7.**	Harris et al. [52]	Minneapolis, MN, area	22 (Not reported)	A series of tomographic images (at approximately 935 × 1001 × 1437 voxels, effective resolution 11.41 × 12.21 × 17.53 mm) were obtained for each specimen. CT Pro software (Nikon Metrology, Leuven, Belgium) was used to reconstruct a 3-dimensional image of each tooth. VG Studio MAX 2.1 software (Volume Graphics GmbH, Heidelberg, Germany) was then used for viewing and measuring	2D &3D	Not reported
**8.**	Filpo-Perez et al.[5]	Brazilian population	100 (Not reported)	50 kV, 800 mA, a rotation step of 0.8, 360of rotation, and an isotropic resolution of 19.6 mm. (NRecon v.1.6.9, Bruker-microCT) was used for reconstruction. CTVol v.2.2.1 and Data Viewer v.1.5 software (Bruker-microCT) were used for visualization and qualitative evaluation	2D & 3D	Ethics committee approval
**9.**	Lamia & McDonald [53]	Not reported	114 (Not reported)	18-µm voxel size, medium resolution, 90 kV, 88-µA intensity, 0.5-mm aluminium filter, integration time of 500 ms, and 30-µm slices. scan times were approximately 3.5 h per sample	3D	Not reported
**10.**	Lee et al. [54]	Korean	37 (Not reported)	Voxel size = 31.8 µm3. 3D modelling software V-works 4.0 (Cybermed Inc., Seoul, Republic of Korea) was used for viewing and analysis	2D & 3D	Institutional Review Board approval
**11.**	Gu et al. [55]	Chinese	45 (Not reported)	Each specimen was scanned along the tooth axis with voxel size of 15 mm or 21 mm by using a micro-CT scanner (Inveon; Siemens Medical Solutions, Knoxville, TN). The data sets were transferred to the Mimics 15.01 (Materialise, Leuven, Belgium) software in DICOM format, and Mimics was used to read and reconstruct the data into 3D images	3D	Medical Ethics Committee
**12.**	Versiani et al. [22]	Brazilian	25 mandibular first molars with MMCs (Not reported)	100 kV, 100 µA and an isotropic voxel size of 9.9 µm. The scanning procedure was performed through 180º rotation around the vertical axis, with a rotation step of 0.4º, using a 0.5-mm-thick aluminium filter. (NRecon v.1.6.9; Bruker-microCT) and (CTAn v.1.14.4; Bruker-microCT) software were used for reconstruction and surface representations of the internal anatomy, respectively	2D &3D	Research Ethics Committee
**13.**	Versiani et al. [56]	Brazilian and Turkish populations	258 Brazilian (136) and Turkish (122), then 48 teeth with MMCs were assessed (Not reported)	100 kV, 100 mA and an isotropic voxel size of 9.9 mm. Scanning procedure was performed through 180˚ rotation around the vertical axis, with a rotation step of 0.4, using a 0.5-mm-thick aluminium filter. (NRecon v.1.6.9; Bruker-microCT) and (CTAn v.1.14.4; Bruker-microCT) software were used for reconstruction and surface representations of the internal anatomy, respectively	Cross and coronal sections of the 3D images	Research Ethics Committee
**14.**	Wolf et al. [57]	Egyptian	118 (Not reported)	Isotropic resolution of 20 mm, 70 kV and 114 mA, resulting in 800–1200 slices per tooth. (VGStudio Max 2.2; Volume graphics, Heidelberg, Germany) was used for reconstructing and viewing the images	3D	Not reported
**15.**	Gu et al. [58]	Chinese	25 (Not reported)	Voxel size of 15 or 21 μm. Mimics 15.01 (Materialise, Leuven, Belgium) software was used for reconstructing and viewing the images	3D	Medical Ethics Committee
**16.**	Keles & Keskin [59]	Turkish	Initially 269 then only 83 roots with Vertucci type II (Not reported)	100 kV and 100 mA. Slices presenting 2000 × 1330 pixel resolution with 10 mm pixel size were obtained from each root by using an 11 MP camera. Scanning was performed at 180 rotations around the vertical axis with a camera exposure time of 1400 ms and a rotation step of 0.4. NRecon software (v. 1.6.4; Bruker-microCT) and CTAn software (v.1.13; Bruker-microCT) were used for the reconstruction and measuring, respectively. Beam-hardening correction of 45%, smoothing of 2, and an attenuation coefficient range of 0–0.06	2D & 3D	Ethical board
**17.**	Keles & Keskin [26]	Not reported	85 mandibular molar teeth with MMCs	100 kV and 100 mA. Slices presenting 2000 X 1330 pixel resolution with 10-mm pixel size were obtained from each root using an 11-megapixel camera. Scanning was performed with 180˚ rotations around the vertical axis with a camera exposure time of 1400 ms and a rotation step of 0.4˚. Data were reconstructed using NRecon software (v. 1.6.4, Bruker-microCT) with a beam-hardening correction of 45%, smoothing of 2, and an attenuation coefficient range of 0–0.06. CTAn and Data Viewer (v.1.5, Bruker microCT) software were used to present the root canal configuration of each root	2D	Not reported
**18.**	Moe et al. [60]	Myanmar	Initially, 181, then 75 were selected (Not reported)	10-μm isotropic resolution, 125 μA, 80 kV, 1-mm aluminium filter, and 0.4° rotation step with 180° rotation. NRecon software v1.6.1 (Bruker MicroCT) and CTAn software v1.14.4 (Bruker MicroCT) were used for reconstruction. CTvol software v2.2.3 (Bruker MicroCT) was used for visualizing the images	2D & 3D	Institutional Review Board
**19.**	Wolf et al. [61]	Not reported	118 (Not reported)	70 kV and 114 μA, resulting in 800–1200 slices per tooth at an isotropic resolution of 20 μm. (VGStudio Max2.2; Volume-graphics, Heidelberg, Germany) was used to be able to differentiate the tooth structures	Axial and coronal sections of 3D images	Not reported
**20. detecti** **21.**	Keles & Keskin [62]	Turkish	Mesial roots of 269 teeth, then only 109 selected (Not reported)	100 kV and 100 mA. Slices presenting 2000 × 1330 pixel resolution with 10-mm pixel size were obtained from each root using an 11-megapixel camera. NRecon software (v. 1.6.4, Bruker-microCT) was used for reconstructing the images with a beam hardening correction of 45%, smoothing of 2, and an attenuation coefficient range of 0 to 0.06. CTAn and DataViewer (v.1.5, Bruker microCT) software were used to reveal the root canal configuration of each root	2D & 3D	Not reported
**22.**	Keles & Keskin [63]	Not reported	269 mesial roots then only 40 (Not reported)	100 kV and 100 mA. Slices presenting 2000 × 1330 pixel resolution with 10 mm pixel size were obtained from each root by using an 11 MP camera. Scanning was performed at 180 rotations around the vertical axis with a camera exposure time of 1400 ms and a rotation step of 0.4˚. NRecon software (version 1.6.4, Bruker-microCT) and CTAn software (version 1. 13, Bruker-microCT) were used for image reconstruction and analysis, respectively	2D & 3D	Ethical board
**23.**	Theye et al. [64]	Skulls from South African	24 (Not reported)	100 kV voltage, 100 mA current, and 2.00 s exposition time per projection, with an isotropic voxel size ranging from 40 to 48 mm. Nikon CT Pro (Nikon Metrology) and VG Studio MAX-3.0 (Heidelberg, Germany) software were used for reconstructing the images and visualization, respectively	2D &3D	Research Ethics Committee
**24.**	Tomaszewska et al. [4]	Not reported	108 (Not reported)	Spatial resolution 13.68 µm per pixel CTVox, CTAn alyser and CTVol (SkyScan®) applications were used for reconstruction and visualisation	3D	Bioethical Commission
**25.**	Marceliano-Alves et al [7]	Brazilian	140 (Not reported)	50 kV, 120 mA, with a rotation step of 0.8, 360° around the vertical axis, and 12.1 µm pixel size, using a 1-mm-thick aluminium filter. NRecon software (v 1.6.1.0; Bruker, Kontich, Belgium). Reconstruction parameters included a 50% beam hardening correction, ring artefact correction of 5 and smoothing of 5. (CTAn v.1.14.4, Bruker-microCT) were used for reconstruction and measuring, respectively	2D & 3D	Ethical committee
26.	Arfianti et al. [65]	Not reported	19 (Not reported)	Resolution, 50 µm (medium); voltage, 130 kV; current, 60 µA; rotation angle, 240°; and time exposure, 295 ms. NRecon and NRecon Server software were used for reconstruction and analysis. DataViewer and Fiji ImageJ software were used for visualising and measuring the parameters, respectively	2D	Not reported
27.	Asijavičienė et al. [66]	Not reported	60 (Not reported)	110 kV, 50 mA, 1-mm aluminium filter, 180° rotation around the vertical axis with rotation step of 0.18 and an isotropic resolution of 22.8 μm. (NRecon v.1.6.9, Bruker-microCT) and CTVol 1.10.1.0 software (Bruker-microCT) were used for reconstruction and volumetric visualisation, respectively	2D &3D	Local ethical committee
28.	Keles et al. [24]	Turkish	Initially 250 then only 30 for bifid and 30 for non-bifid, (by using [G*Power 3.1])	100 µA, 100 kV, 180° rotation with a step of 0.4°, frame average of 3 and 1,400 ms of exposure duration. Pixel size of 10 µm. Data were reconstructed (NRecon v. 1.7.4.2 software; Bruker-microCT) with ring artefact (5), beam-hardening (45%), and smoothing (2) corrections using an attenuation coefficient ranging from 0 to 0.06. CTAn v.1.18.8 software (Bruker-microCT) and CTVol v. 2.3.2.0 software (Bruker microCT) were used for 3D reconstruction and qualitative analysis	2D & 3D	Ethics committee
29.	Mazzi-Chaves et al. [67]	Brazilian	50 (Not reported)	Voxel size of 26.70 μm. The scanning parameters used were 50 kV, 800 μA, 180° rotation around the vertical axis, rotation step of 1°, and a 0.5 mm-thick aluminium filter, rendering a scan time of 25 min, approximately. NRecon v.1.7.1.0 software (Bruker-microCT, Kontich, Belgium) ring artifact reduction of 5, beam hardening correction of 40%, smoothing of 3, and an attenuation coefficient between 0.001 and 0.15. DataViewer v.1.5.4.0 software and (Bruker-microCT, Kontich, Belgium) were used for reconstruction and measuring, respectively. CTAn v.1.17.7.2 + software (Bruker microCT, Kontich, Belgium) for generating 3D models	3D	Research Ethics Committee
30.	De-Deus et al. [11]	Brazilian subpopulation	120 ([G*Power 3.1] software)	14.25 μm (pixel size), 70 kV, 114 mA, 180° rotation around the vertical axis, rotation step of 0.7°, camera exposure time of 250 ms, frame average of 4, using a 1-mm-thick aluminium filter. (NRecon v. 1.7.1.6; Bruker-microCT) was used for reconstruction with beam hardening (35 to 45%), ring artefact correction (3 to 5), and contrast limits (0 to 0.05). DataViewer v.1.5.6 software (Bruker-microCT) was used for qualitative and quantitative analysis	2D & 3D	Local ethical committee
31.	Fu et al. [36]	Chinese	136 (Not reported)	90 kV/88 mA with an isotropic voxel size of 30 mm. Scanning was performed by 500 projections per 180, camera exposure time of 500 ms	3D	Ethics committee
NO	**Studies**	**Type of study**	**Type of analysis**	**Classification used**	**Calibration and assessment reliability**	
1.	Mannocci et al. [47]	Retrospective	Quantitative	N/A	Two examiners	
2.	Iwaka et al. [48]	Prospective	Quantitative	N/A	Not reported	
3.	Gu et al. [49]	Prospective	Quantitative and qualitative	Weller system for isthmus classification	Agreement had been achieved by three observers	
4.	Gu et al.[12]	Prospective	Quantitative and qualitative	Vertucci system for canal configuration	Not reported	
5.	Gu et al. [50]	Prospective	Quantitative	N/A	3 times repeated measurements	
6.	Fan et al. [51]	Retrospective	Quantitative and qualitative	Fan classification for isthmus	Not reported	
7.	Harris et al. [52]	Retrospective	Quantitative	Vertucci	Not reported	
8.	Filpo-Perez et al.[5]	Retrospective	Quantitative and qualitative	Vertucci system for canal configuration	Not reported	
9.	Lamia & McDonald [53]	Retrospective	Quantitative	N/A	Two examiners	
10.	Lee et al. [54]	Retrospective	Quantitative	N/A	Not reported	
11.	Gu et al. [55]	Prospective	Quantitative	N/A	Intra- and inter-observer agreement was estimated on 8 specimen (1 specimen for each root form), and each specimen was measured twice. Intraclass and one-way random effects model were calculated. The interobserver agreement in RSA was higher in both examiners, with an ICC of 0.999 (95% CI: 0.993, 1.000) (*p* = 0.000) in examiner 1 (Gu Y) compared to correlation coefficients (ICC) based on 0.999 (95% CI: 0.994, 1.000) (*p* = 0.000) in examiner 2 (Zhu Q). The ICC for inter-observer agreement was 0.994 (95% CI: 0.973, 0.999) (*p* = 0.000)	
12.	Versiani et al. [22]	Retrospective	Quantitative	N/A	Not reported	
13.	Versiani et al. [56]	Retrospective	Quantitative and qualitative	Pomeranz system for middle mesial canal	Not reported	
14.	Wolf et al. [57]	Retrospective	Quantitative and qualitative	Four-digit system for canal configuration	Not reported	
15.	Gu et al. [58]	Retrospective	Quantitative	N/A	Not reported	
16.	Keles & Keskin [59]	Retrospective	Quantitative	Vertucci system for canal configuration	Not reported	
17.	Keles & Keskin [26]	Retrospective	Quantitative and qualitative	Pomeranz system for middle mesial canal	Not reported	
18.	Moe et al. [60]	Retrospective	Quantitative and qualitative	Weller for isthmus classification	Not reported	
19.	Wolf et al. [61]	Retrospective	Quantitative and qualitative	N/A	Not reported	
20.	Keles & Keskin [62]	Retrospective	Quantitative	N/A	Not reported	
21.	Keles & Keskin [63]	Retrospective	Quantitative and qualitative	N/A	Not reported	
22.	Theye et al. [64]	Retrospective	Quantitative	N/A	Not reported	
23.	Tomaszewska et al. [4]	Prospective	Quantitative	Vertucci for canal configuration	Two researchers did the measurements, and the 3^rd^ one averaged them	
24.	Marceliano-Alves et al.[7]	Retrospective	Quantitative and qualitative	Vertucci for canal configuration. Hsu & Kim for isthmus	Not reported	
25.	Arfianti et al. [65]	Retrospective	Qualitative	N/A	Not reported	
26.	Asijavičienė et al. [66]	Retrospective	Quantitative and qualitative	Fan classification for isthmus	Not reported	
27.	Keles et al. [24]	Retrospective	Quantitative and qualitative	N/A	Not reported	
28.	Mazzi-Chaves et al. [67]	Retrospective	Quantitative	Pucci & Reig and AAE for RCC	Not reported	
29.	De-Deus et al. [11]	Retrospective	Quantitative and qualitative	N/A	Not reported	
30.	Fu et al. [36]	Retrospective	Qualitative	N/A	Not reported

**Table 3:** Characteristics of the included studies: objective(s) of interest

**Studies**	**Canal morphology (3D and/or 2D)**	**Apical morphology**	**Accessory canals**	**Isthmuses**	**Dentine thickness**	**Root morphology**	**Pulp chamber**	MMCs
1. Mannocci et al. [47]				**✓**				
2. Iwaka et al. [48]							**✓**	
3. Gu et al. [49]				**✓**				
4. Gu et al. [12]	**✓**		**✓**				**✓**	
5. Gu et al. [50]	**✓**							
6. Fan et al. [51]				**✓**				
7. Harris et al. [52]	**✓**		**✓**	**✓**	**✓**		**✓**	
8. Filpo-Perez et al. [5]	**✓**							
9. Lamia & McDonald [53]	**✓**							
10. Lee et al. [54]	**✓**				**✓**			
11. Gu et al. [55]						**✓**		
12. Versiani et al. [22]		**✓**						
13. Versiani et al. [56]							**✓**	**✓**
14. Wolf et al. [57]	**✓**	**✓**	**✓**					
15. Gu et al. [58]						**✓**		
16. Keles & Keskin [59]	**✓**							
17. Keles & Keskin [26]								✓
18. Moe [60]	**✓**		**✓**	**✓**			**✓**	**✓**
19. Wolf [61]		**✓**						
20. Keles & Keskin [62]		**✓**						
21. Keles & Keskin [63]				**✓**				
22. Theye et al. [64]						**✓**		
23. Tomaszewska et al. [4]	**✓**							
24. Marceliano-Alves et al. [7]	**✓**		**✓**	**✓**				
25. Arfianti et al. [65]	**✓**							
26. Asijavičienė et al. [66]		**✓**	**✓**	**✓**				
27. Keles et al. [24]		**✓**	**✓**	**✓**	**✓**			**✓**
28. Mazzi-Chaves et al. [67]	**✓**							
29. De-Deus et al. [11]					**✓**	**✓**	**✓**	
30. Fu et al. [36]	**✓**							

The reviewers adopted the AQUA tool to assess the quality of the selected studies that dealt with anatomical features of the mandibular first molar. The quality assessment process was performed by two independent reviewers (HMA and HMAA). AQUA tool was proposed to assess the potential risk of bias amongst included studies [68]. It is composed of five domains: 1. Objective(s) and Subject Characteristics; 2. Study Design; 3. Methodology Characterization; 4. Descriptive Anatomy; and 5. Results Reporting. Each domain consists of a set of signaling questions to assist in evaluations and judgements about risk of bias pertaining to the domain. The signaling questions are answered as “Yes”, “No”, or “Unclear”. For these signaling questions, “Yes”, “No”, and “Unclear” indicate low, high, and unclear risk of bias, respectively. On the other hand, the risk-of-bias question is judged as “Low”, “High”, or “Unclear”. If all signaling questions for a domain are answered “Yes”, then risk of bias can be judged “Low”. If any signaling question is answered “No” or “Unclear”, this indicates a potential risk of bias. A consensus regarding this should be reached between the reviewers. The “Unclear” option should be used only when the reported data are insufficient to allow for a clear judgment.

The overall risk of bias for each study was evaluated based on how many of the domains were met (i.e., based on the results of the ROB assessment). Accordingly, they were classified, as described in the study of Borges et al. [69] (with modification), into the following categories:

- low risk of bias (i.e., studies that had a low risk of bias in at least 4 of the ROB domains).
- moderate risk of bias (i.e., studies that had between 2 and 4 of the ROB domains scored with ''low risk of bias'').
- high risk of bias (i.e., studies that had at least 4 of the ROB domains scored with "potential risk of bias").

## Results

### Study identification

In the first phase, the search query yielded 1358 articles from the three databases specified. After deleting the duplicates, there were 921 articles. A total of 831 articles were excluded in the second phase of screening the titles. Following the third phase of abstract reading, based on the exclusion criteria, another 56 articles were excluded (Fig. 1). A total of 34 articles were eligible for inclusion. In the fourth phase, based on full-text reading, 4 studies [70–73] were excluded from the final set due to the lack of specific details about mandibular first molars, as they either presented details about first and second mandibular molars collectively or about mandibular molars in general. A final set of 30 articles were included for analysis (Fig. 1 and Tables 2, 3).

### Study risk of bias assessment

**Fig. 2:**
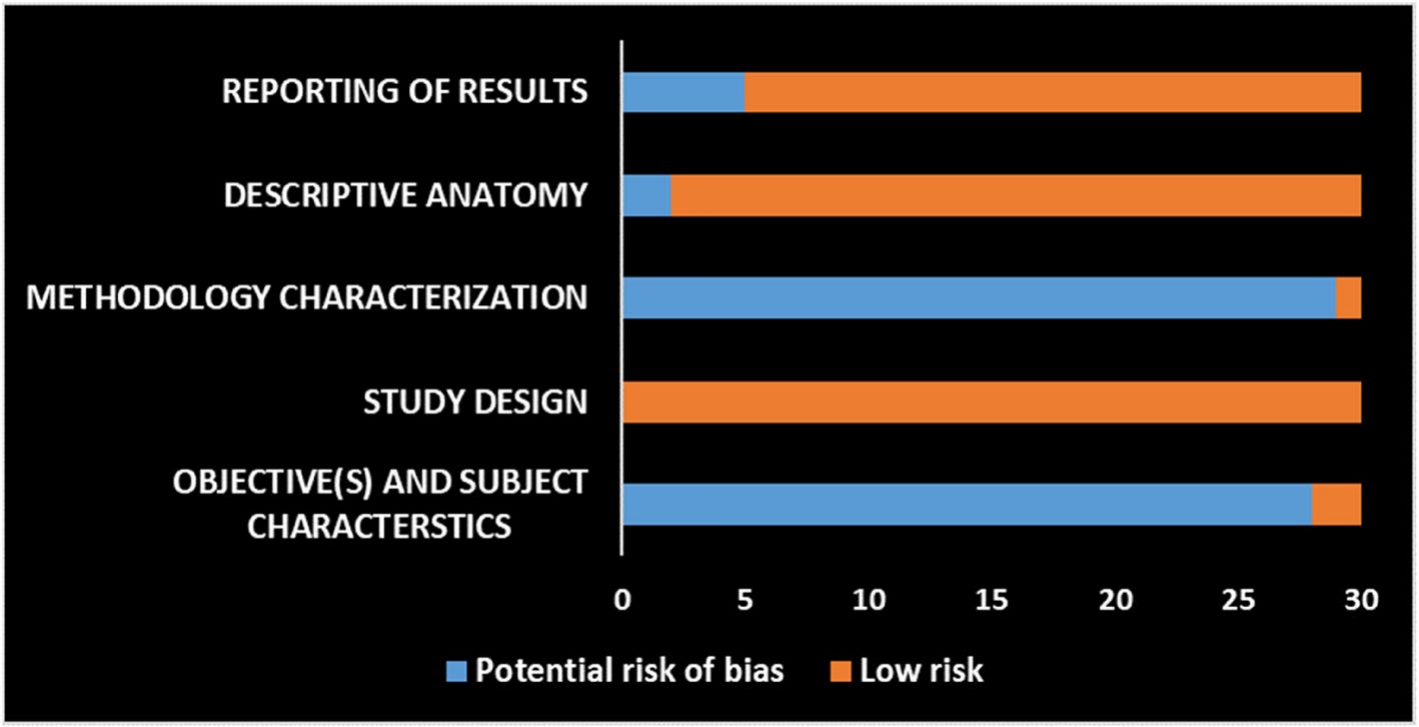
Risk of bias graph: Reviewers’ judgements about each risk of bias domain (AQUA tool domains)

The results of risk of bias assessment of the selected studies are shown in Fig. 2. The vast majority of the included studies (28 out of 30) had a potential risk of bias in domain one (Objective(s) and Subject Characteristics). This is mainly due to the lack of any elaboration or clarification about the method of sample size calculation. All the included studies had low risks of bias in domain two (Study Design). All studies (except one) showed a potential risk of bias in domain three (Methodology Characterization), principally due to two reasons. First, the medical speciality and experience of the individuals who conducted each part of the study were rarely mentioned. Second, absence or poor reporting of the measures that had been taken to reduce the inter and intra-observer variability. Domain four (Descriptive Anatomy) was judged mostly free of risk of bias, except for studies in which the presented figures (images, illustrations, diagrams, etc.) were either unclear or not understandable. Domain five (Reporting of Results) reported a low potential risk of bias (25 out of 30), except for those studies that adopted inadequate or inappropriate statistical analysis.

The overall ROB assessment indicated that the vast majority of the included studies had a "moderate" risk of bias (27 studies), only two studies had a "low" risk of bias, and a solitary study was referred to as having a "high" risk of bias [65]. As a result, no sensitivity test was performed, as none of the studies included in the meta-analysis was considered to be at high risk of bias.

### Meta-analysis

**Fig. 3:**
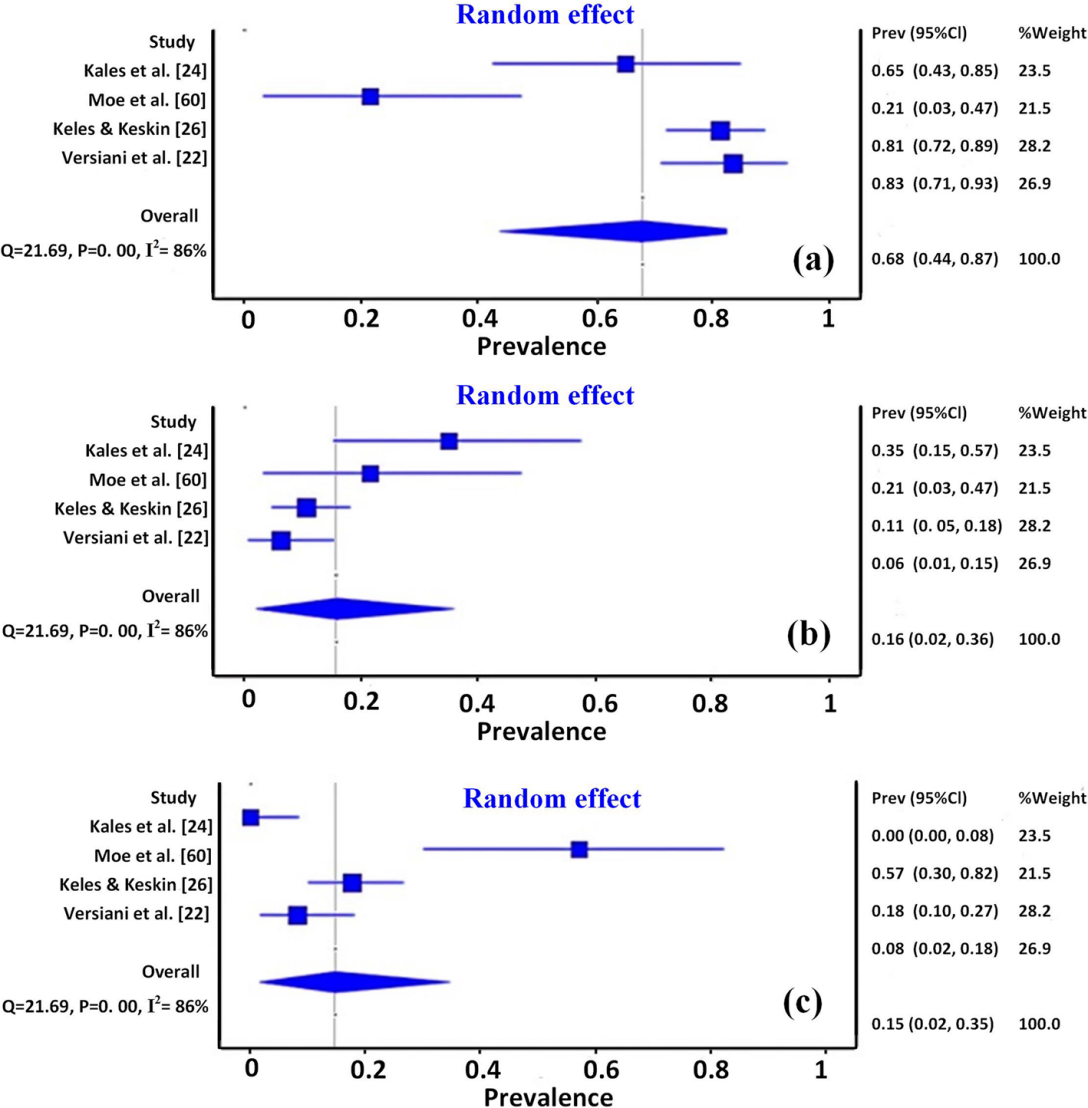
Forest plots of meta-analysis for middle mesial canals types, (**a**) Confluent, (**b**) Fin, and (**c**) Independent

**Fig. 4:**
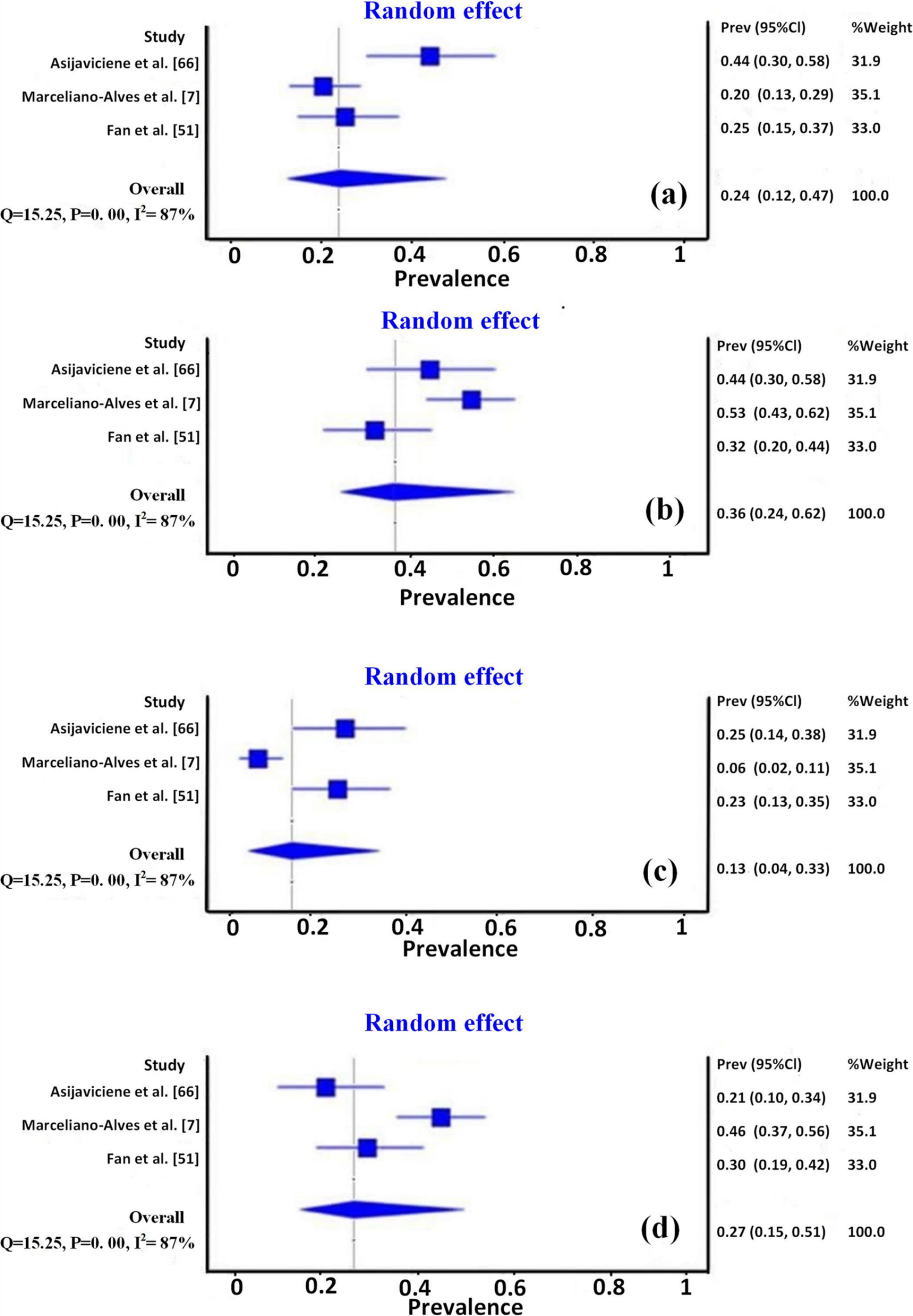
Forest plots of meta-analysis for Fan's isthmus types, (**a**) type I, (**b**) type II, (**c**) type III, and (**d**) type IV

To allow comparison amongst studies, the important results of the objective/s in each study were extracted, summarised, and presented in tables or charts, separately. The results of the meta-analysis for MMCs configurations and isthmus types are presented in forest plots (Figs. 3, 4). The diamond shapes in the forest plots, representing the overall effect of all studies that dealt with that objectives, pass over the no-effect line, indicating no significant difference. Heterogeneity was evaluated using the I^**2**^ test, which analyses the proportion of total variability between studies explained by heterogeneity. A value above 60% was considered to be substantial heterogeneity. In this context, the meta-analysis result also showed a high level of heterogeneity (I^**2**^ = 86% and 87% for the studies related to MMCs and Fan's isthmus types, respectively), which can be explained by the different anatomical variations in different population groups, sample size as well as different setting parameters for micro-CT scanning.

The meta-analysis results for studies that focused on the prevalence of different types of main canal configurations (according to Vertucci) are presented in Table 4. Those results (pooled prevalence) showed a wide variety in the reported configurations in the mandibular first molar. Type IV (0.587%) (95%CI: 0.186—0.627) and type I (0.830%) (95%CI: 0.623—0.880) configurations were the most prevalent in mesial and distal root canals, respectively.

**Table 4:** Prevalence of different Vertucci types of canal configuration in mesial and distal root canals of mandibular first molar

**Types of canal configuration (Vertucci)**	**Mesial Root**	**Distal Root**
	**Pooled prevalence: %** **(95% CI)**	**Pooled prevalence: %** **(95% CI)**
Type I	0.132 (0—0.360)	0.830 (0.623—0.880)
Type II	0.110 (0—0.329)	0.034 (0—0.095)
Type III	0.049 (0—0.227)	0.015 (0—0.060)
Type IV	0.587 (0.186—0.627)	0.024 (0—0.077)
Type V	0.141 (0—0.373)	0.061(0.003—0.150)
Type VI	0.073 (0—0.271)	0.027 (0—0.082)
Type VII	0.029 (0—0.183)	–
Type VIII	0.034 (0—0.195)	–

## Discussion

Since the areas of focus in this review are the objectives of interest and certain study specifications (e.g., sample size, population, and micro-CT setting specifications), as well as most of the anatomy studies included many objectives at a time, it was not possible to perform a taxonomy based on different objective domains. Within these limitations, the main body of the discussion section is essentially created based on discussing the highlighted results, figuring out the root and canal anatomic variability of this tooth type, identifying the gaps and suggesting future directions, and transferring the generated outcome into the clinical implications.

### Root morphology

#### Root length

Anatomical changes in the roots of human teeth are common, and their rate of occurrence, distribution pattern, and morphological characteristics might differ among ethnic groups. Moreover, the data derived from one ethnic group may be inapplicable to another during dental treatment [58]. Knowing the root length and curvature is important to avoid potential damage during root canal treatment [50].

Such anatomical traits could be affected by the population of interest, age, and even methodology. For instance, the mean root length of the two-rooted mandibular first molar was around 13.16 ± 1.24 mm in the Chinese population (Table 5) [58]. However, Theye et al. [64] reported different results, namely a mean length of 9.94 ± 0.85 mm. They used a different approach to determine root length by measuring the distance between the deepest landmark of the interradicular bone (I) and the centre of root apex in skulls' jaws bones of unclaimed bodies of African ancestry. Additionally, different ages have been tested, ranging from 22 to 76 years, with a slightly older mean age (40 years). However, due to the different approach used for measuring the root length and the presence of the cortical and interradicular jaw bones, which might compromise the procedure, it is not surprising to see such inconsistent results.

**Table 5:** Summary of root length results in mandibular first molar

**Studies**	Whole tooth/ root (N)	**Objective Description**	**Mean ± SD (Range)**
Gu et al*.* [55]	**Whole tooth** (25)	The length of the root was measured as the vertical distance from the anatomical root apex to the highest level of the CEJ	13.17 ± 1.24
Gu et al*.* [58]	**Whole tooth** (25)	Root length: The vertical distance between the highest level of the CEJ and the root tip along the long axis of the tooth	13.16 ± 1.24 mm
Theye et al*.* [64]	**Whole tooth** (61)	Root length: the distance between deepest landmark of interradicular bone *(I) and root apex centroid	9.94 ± 0.85 (8.48—11.07)

(*N*) Number of samples, *(I): the deepest landmark of interradicular bone, *MR* Mesial root

#### Root dentine thickness

An accurate understanding of the canal position and surrounding dentine morphology is required for successful root canal therapy. In mesial roots of permanent mandibular molars, Abou-Rass et al. [74] have labelled the 'danger zone' because of anatomical features such as a curved canal and deep radicular grooves. It renders the root more vulnerable to fractures and strip perforations when the canal is over-prepared and transported.

In either root of a two-rooted permanent mandibular first molar, the danger zone could be expected anywhere along the furcal aspect (the aspects of the mesial and distal roots being directed toward the root's furcation). Thus, the entire furcal aspect of the root might be considered a ‘danger zone’ (area at risk for strip perforation). Consequently, in order to reduce the chance of strip perforation, care must be taken while conducting root canal instrumentation. This can be achieved by using anti-curvature motion, avoiding doing extra flaring, the use of modern nickel-titanium rotary and reciprocating instruments, and sequential preparation techniques that maintain the original canal anatomy with less canal transportation and better centring ability [52, 54]. Table 6 shows a summary of root dentine thickness results in the mandibular first molar. However, high percentages of the thinnest dentine in the mesial root have been reported to be directed mesially at different levels of the root length. In 48% of samples, it was directed mesially, especially in the apical third [54]. In another work, the amount of mesially directed thinnest dentine was notably less than that reported in the study of Lee et al. [54], with no significant difference – except at the furcation area – between bifid and non-bifid roots [24]. However, this difference might be attributed to the methodology, as Lee et al*.* [54] studied the whole root length, whereas Keleş et al*.* [24] evaluated only up to 7 mm apical to the root furcation. However, generating a 3D colour-coded map for the root dentine thickness could be an effective illustrative tool for qualitative comparison [11].

**Table 6:** Summary of root dentine thickness results in mandibular first molar

**Studies**	Whole tooth/ root (N)	Approach	DT Mean mm (SD)	Measuring Position	Direction of DZ
Harris et al*.* [52]	Whole tooth (22)	Using the imaging software, the entire mesial root was sectioned from the root apex coronally in 0.5-mm increments for the first 6 mm and in 1.0-mm increments from the 6-mm point to the level of the furcation. The first 6 mm of the distal root was also sectioned in 0.5-mm increments coronally from the apex	DZ in MR = 1.28 mm DZ in DR ranged from 0.25 mm (at the 0.5-mm level from the apex) To 1.47 mm (at the 5.0-mm level)		toward furcation
Lee et al*.* [54]	MR (37)	3D surface models were re-sliced at 0.1-mm intervals perpendicular to the central axis through the whole length of the canal	1.16 (± 0.37) 1.00 (± 0.28) 1.86 (± 0.60) Thinnest dentine = 0.88 (± 0.26)	Mesially Distally Laterally (buccal and lingual)	MB (65%) disto-inside, and (35%) mesially ML (72%) disto-inside, and (28%) mesially
Keles & Keskin [24]	MR 30 bifid and 30 non-bifid	3D map of dentine thickness was obtained and colour-coded. The DZ was measured at each 1 mm slice from 1 to 7 mm level apical to the furcation	bifid = 1.16 ± 0.16 bifid = 1.08 ± 0.18 non bifid = 1.18 ± 0.15 non bifid = 1.12 ± 0.18	Mesially Distally Mesially Distally	In the mesial direction, it was ranged from 0.49 to 1.88 mm (bifid roots) and from 0.43 to 1.85 mm (non-bifid roots), in the distal direction it varied from 0.32 to 2.14 mm (bifid roots) and from 0.40 to 1.92 mm (non-bifid roots)
De-Deus et al*.* [11]	MR (28)	3D models of root surfaces and canals were created and the central axis obtained. DZ was estimated on re-sliced planes made perpendicular to the central axis of each canal at 0.1 mm for the whole length	DZ = 0.86 (± 0.15)		60.7% and 71.4% are located distally in group *I and *II respectively 39.2% and 28.6% are located mesially in group *I and *II respectively

(*N*) Number of roots or teeth, (*SD*) Standard deviation, *DT* Dentine thickness, *DZ* Danger zone (thinnest dentine), *MR* Mesial root, *DR* Distal root

### Root canal morphology

In the current review, the most frequently studied aspect is the root canal morphology, including the assessment of the 3D configuration of the canal system, the number of canals in the 3D reconstructed images or 2D sections, canal taper, canal curvature, and 2D parameters (perimeter, area, major and minor dimensions, roundness, aspect ratio) in human permanent mandibular first molars.

### Root canal configuration

#### Configuration of the main canal

The main root canal is defined as a passage or channel in the root of the tooth extending from the most apical portion of the pulp chamber (i.e., root canal orifice) to the major apical foramen [75]. This definition describes the start and end points of the main canal regardless of its configuration, differentiating it from accessory canal and other entities. The root canal configuration is the course of the root canal system that begins at the orifice and ends at the canal terminus [76]. This definition describes the course of that main canal (i.e., how many canals are there and whether they are splitting or joined etc.).

Among the studies included in this systematic review, Vertucci classification system was used most frequently to describe the root canal configuration, followed by the four-digit system. Although those systems represent the canal configuration usefully, noticeably higher percentages of non-classifiable configurations have been reported in micro-CT studies. (Table 7).

**Table 7:** Summary of main canal configuration results in mandibular first molar

**Studies**	Whole tooth/ root (N)	Population	system	Configuration %		N.C %
				**MR**	**DR**	
Gu et al. [12]	Whole tooth (25)	Chinese	Vertucci	Type I 8% Type II 12% Type IV 64% Type V 8%	Type I 72% Type II 4% Type IV 4% Type V 4%	
Harris et al. [52]	Whole tooth (22)	Minnesota, USA	Vertucci	Type I 9.09% Type II 0% Type III 4.54% Type IV 4.54% Type V 27.27% Type VI 9.09% Type VII 4.54% Type VIII 0%	Type I 81% Type II 0% Type III 4.54% Type IV 0% Type V 4.54% Type VI 0% Type V 4.54% Type VIII 0%	18% of MRs
Filpo-Perez et al. [5]	DR (100)	Brazilian	Vertucci		Type I 76% Type II 3% Type IV 1% Type V 7%	13%
Lamia & McDonald [53]	MR (114)	N/A	Vertucci	Type IV only 50.8%		
Wolf et al. [57]	Whole tooth (118)	Egyptian	Four-digits	2–2-2/2 31.4% 2–2-1/1 15.3% 2–2-2/3 11.9% 2–2-1/2 7.6% Others 0.8- 4.2%	1–1-1/1 58.5% 1–1-1/2 10.2% 1–1-2/2 4.2% 1–1-1/3 3.4%	
Marceliano-Alves et al. [7]	MR (104)	Brazilian	Vertucci	Type I 11.5% Type II 16.3% Type III 5.8% Type IV 46.2% Type V 2.9% Type VI 8.7% Type VII 1% Type VIII 7.7%		

*N* Number of teeth or roots, *N/A* Not applicable, *N.C* Non classifiable, *MR* Mesial root, *DR* Distal root

#### Root canal configurations in the mesial root

The most frequent finding was two canals in the mesial roots of mandibular first molars (Table 7). Different representations of two canals have been reported in the literature. It could be two separate mesial canals [(i.e., type IV); [7, 57]] or start with a single canal and then divide into two canals [Vertucci type V;[7]]. Particularly with the advancement in micro-CT scanners that provide smaller voxel sizes and consequently higher resolution, intriguing findings have been achieved and new concepts have been adopted. Many of the mesial and distal root canal configurations have been reported as ‘non-classifiable’ using the Vertucci classification system, especially in micro-CT-based studies [5, 6, 77]. However, a wide variety of mesial canal configurations have also been reported, indicating the complexity and variability of the mesial canal anatomy compared with the distal canal anatomy (Table 7).

#### Root canal configurations in the distal root

Regardless of the classification system being used and the population being examined, the presence of a single canal was the most frequently reported configuration in the distal root of the two-rooted mandibular first molar. In addition to some differences in the percentage of a single canal in different populations, 3 or 4 distal canals have been reported in Brazilians [5] but not in Egyptians [57]. This indicates different anatomical variations among ethnic groups.

#### Configuration of additional or middle mesial canals

A number of publications have described this anatomical variant [24, 26, 60], referred to as the mesio-central canal, the third mesial canal, the intermediate canal, the mesial accessory canal, and the middle mesial canal (MMC) [22]. Although the authors did not prove hypotheses by experiments, they speculated that as the root forms, the connective pulp tissue is pressed by the deposition of secondary dentine, forming vertical dentine partitions within the root canal space, resulting in the formation of three mesial root canals [78].

MMCs have been classified into independent, fin, and confluent morphologies [79]. However, confluent anatomy could be subdivided as with or without an isthmus [22]. Because the MMCs could be found in a developmental groove between the mesial canals, extensive exploration of this groove is essential [22, 60]. Keleş and Keskin [26] found that no troughing was required to locate 77.41% of the MMC orifices as they were located at the cemento-enamel junction (CEJ). However, 1- and 2-mm troughing depth would be useful in locating the orifices in 5.38% and 9.69% of the specimens, respectively. Moreover, 7.52% of the MMCs were inaccessible even by deep troughing as their orifices were seated deeper than 2 mm from the CEJ [26].

In the literature, higher incidences have been reported in different populations, with a most predominantly confluent type (Table 8) [22, 24]. Along its course, the MMC is likely to be closer to lingual rather than buccal mesial canals. However, the fin configuration was the most frequent in the Burmese population [60]. A meta-analysis was performed for this section. The forest plots (Fig. 3) showed a high level of overall heterogeneity (I^**2**^ value was 86% for confluent, fin, and independent type). These inconsistent results might be attributed to the different study designs, different populations, and strict criteria of sample selection. For instance, MMCs were studied only in mesial roots with Vertucci type IV in the study by Moe et al. [60].

**Table 8:** Summary of middle mesial canals (MMCs) results in mandibular first molar

**Studies**	Whole tooth/ root (N)	Total incidence n (%)	MMCs Configuration type			
			**Independent**	**Fin**	**Confluent**	**Double MMC**
Versiani et al*.* [22]	MR (48)	48 (18.6%)	3 (6.30%)	4 (8.30%)	14 (29.20%) with isthmus 26 (54.10%) without isthmus	1 (2.10%)
Moe et al*.* [60]	MR (75)	14 (18.7%)	21.4%	57.1%	21.4%	
Keleş A, Keskin [26]	MR (85)		At CEJ = 5 (5.3%) 1 mm = 0 (0%) 2 mm = 0 (0%) ≥ 2 mm = 4 (4.3%) Total = 9 (9.6%)	At CEJ = 10 (10.7%) 1 mm = 2 (2.1%) 2 mm = 3 (3.2%) ≥ 2 mm = 0 (0%) Total = 15 (16.1%)	At CEJ = 57 (61.2%) 1 mm = 3 (3.2%) 2 mm = 6 (6.4%) ≥ 2 mm = 3 (3.2%) Total = 69 (74.1%)	
Keles et al*.* [24]	MR (30) bifid (30) non-bifid	20 (66.6%) 10 (33.3%) in bifid 10 (33.3%) in non-bifid	5 (16.6%) in bifid 2 (6.6%) in non-bifid	0 cases in both groups	8 (26.6%) without isthmus in non-bifid 5 (16.6%) without isthmus in bifid	

*MMCs* Middle mesial canals, *CEJ* Cementoenamel junction

### Root canal shape

A disparity has been reported between the bucco-lingual and mesio-distal dimensions of the canals in mesial and distal roots, showing that those canals were not truly round [52]. Overall, at the apical 3-mm level, the values of 2D parameters increased considerably. At the 1-mm level, the predominance of oval canals was greater; however, at the 5-mm level, the prevalence of long oval and flattened canals was more remarkable [5, 59]. However, a slight difference in the frequent cross-sectional patterns of root canals could be noticed [65]. Thus, based on the outcomes of these studies, it can be hypothesised that regardless of the root position (mesial or distal), the number of the root canals, and configuration patterns, the cross-sectional canal shape changes from oval to long oval in the apico-coronal direction.

### Root canal curvature

The MB canal had a larger curvature than the ML canal; it was largest in the apical portion, followed by the coronal regions, and straighter in the middle third [50, 54]. The root canal curvature of permanent mandibular first molars was assessed in 3D views in one study [36], and the authors developed a new minimally invasive access technique. The landmarks were positioned more mesiobuccally in relation to the centre of the molars' occlusal plane. The maximum curvature of coronal root canals in the axial direction was determined as follows: in three-canaled, two-rooted mandibular first molars, the average curvature angles were 23°, 25°, 11° for the MB, ML, and distobuccal (DB) canals, respectively. In four-canaled, two-rooted teeth, they were 23°, 25°, 12°, and 16° for MB, ML, DB, and distolingual (DL) canals, respectively [36]. Based on the aforementioned canal curvature outcomes, to overcome potential file damage or canal mishaps, attention and care must be taken while preparing the mesial canals, especially at the apical third.

### Inter-orifice distance

**Fig. 5:**
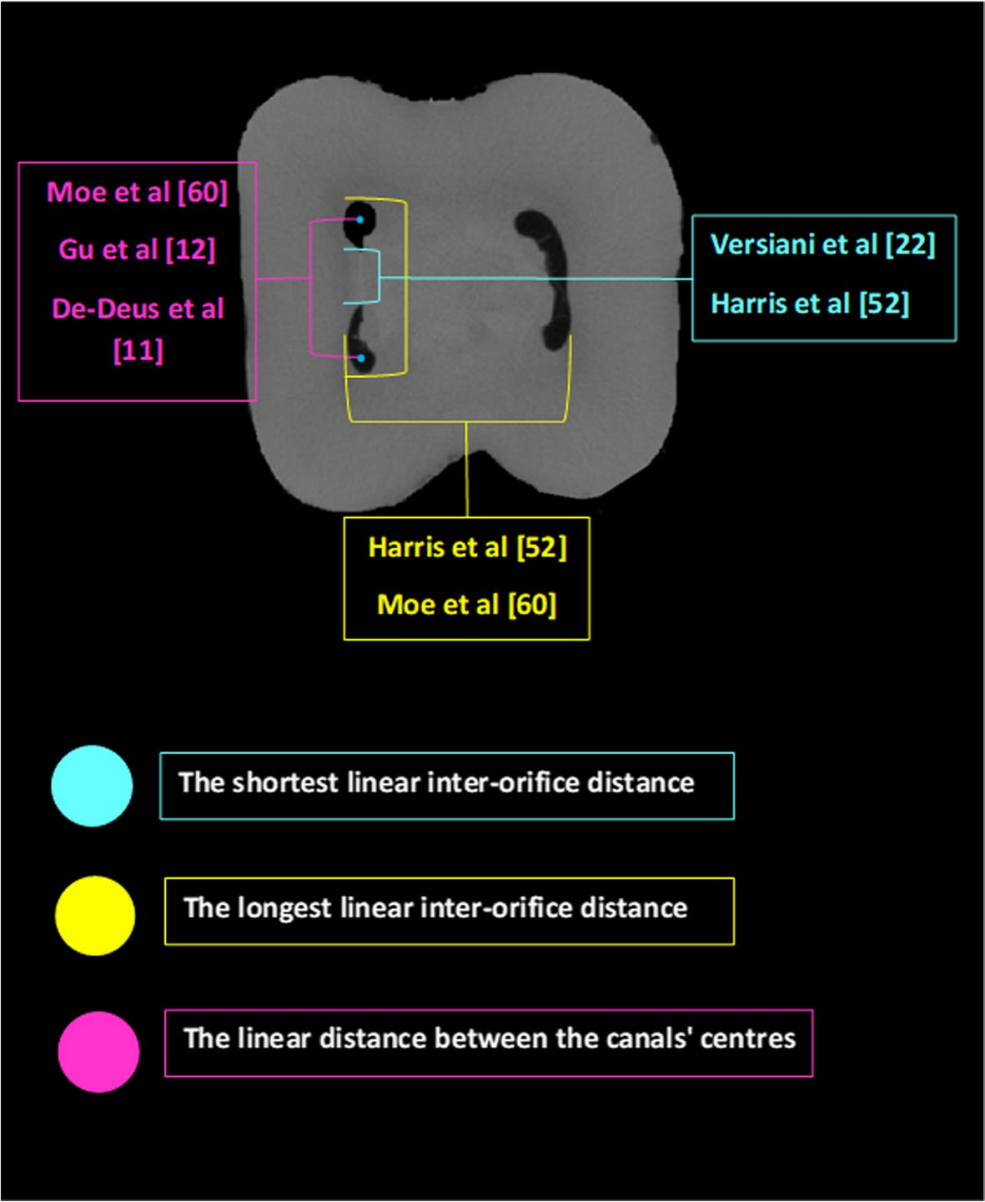
Different approaches for measuring the inter-orifices distances

It is not possible for the clinician to measure or even observe the inter-orifice distance on a 2D radiograph unless there is a CBCT image. Knowledge about this distance could be helpful in producing a conservative yet convenient endodontic access cavity. Geometric data on the pulp floor can be useful for locating the canal orifice. Being unaware of these anatomical features may lead to treatment failure [12]. In this context, rather conflicting results of mean inter-orifices distances have been reported in the literature. The difference in the evaluation procedures can justify these contradictory results. De-Deus et al. [11] recorded this distance at the lowest level of the CEJ on the crown's buccal aspect, which is not identical to the level of 1.5 mm coronal to the furcation adopted in other studies [22, 60]. The sample size and racial factors might also have had an impact on the results. Moreover, different approaches have been adopted in the literature to measure the inter-orifice distance (Fig. 5). While some studies have adopted the closest linear inter-orifice distances [11, 56], others have adopted the longest linear inter-orifice distances [52]. Moreover, the linear distances between the canals' centres have also been adopted [12, 60]. These findings could aid clinicians in detecting a missing canal with a calcified orifice, reduce the time and effort of searching a canal orifice, and reduce the risk of perforation, saving the sound tooth structure. Table 9 illustrates the results of micro-CT studies involved in this review that evaluated the inter-orifice distances in the mandibular first molar.

**Table 9:** Summary of inter-orifices distances results of the mandibular first molar

**Studies**	Whole tooth/ root (N)	IOD Definition	Level	Values (mm) MEAN ± SD (Range)
Gu et al. [12]	Whole tooth/ (25)	length of a line between centre points of two orifices	Pulp chamber floor	MBO-MLO = 2.35
Harris et al. [52]	Whole tooth (22)	1. The shortest linear distance between canals' orifices 2. The longest linear distance between canals' orifices	1.5 mm coronal to the furcation	Longest MB-ML = 3.09 Shortest MB-ML = 1.43 Longest DB-DL = 1.98 Longest M-D = 4.35
Versiani et al*.* [56]	MR (258)	Distance between the mesial canals' orifices	1.5 mm coronal to the furcation	MMC-MLC 1.34 ± 0.74 (0.45–3.92), and MMC-MBC 1.35 ± 0.74 (0.35–3.38)
Moe et al*.* [60]	MR (75)	Distance between the centres of the MB and ML canals	at 1.5 mm coronal to the furcation	2.60 ± 0.41 (1.69–3.48 mm)
De-Deus et al*.* [11]	MR (28)	The linear distance between (MB) and (ML) canal orifices' centres	At the lowest level of the CEJ on the buccal aspect of the crown	MB-ML 3.76 ± 0.89 (2.36–5.58) in grp I 4.49 ± 0.75 (3.28–6.08) in grp II

*IOD* Inter-orifice distance, *CEJ* Cementoenamel junction, *MR* Mesial root, *grp I* Group 1—root length between 8 and 9.6 mm, *grp II* Group 2—root length between 11.5 and 13.1 mm, *MBO* Mesiobuccal orifice, *MLO* Mesiolingual orifice, *MLC* mesiolingual canal, *MBC* mesiobuccal canal

### Apical canal morphology

The instrumentation and filling of root canals are heavily reliant on an accurate anatomical understanding of the apical area. Apical constriction (minor apical foramen), major foramen, and anatomical apex are the three anatomical components in the root apex that can be used to describe apical morphology.

As for the distance between the major apical foramen and anatomical apex (MAF-apex), Keleş and Keskin [63] found that the total average distance was 0.85 mm in mesial root canals (Table 10). Nevertheless, compared with MB and ML root canals, MMCs demonstrated the highest deviation from the anatomical apex (*p* < 0.05; [63]. Of note, the deviation of the major foramen of the MMC was > 2 mm in six cases, going up to 5.67 mm [63]. Moreover, it could reach 6.2 mm [22]. So, whatever the working length of the MB and ML canals is, it might not be applicable to the MMC.

**Table 10:** Summary of apical anatomy results in the mandibular first molar

**Studies**	Sample size	Apical features			
		**MAF- Apex**	**MAF- ApC**	**AF NO**	**ApC dimensions**
Wolf et al. [57]	118			**Number of accessory apical foramen only** **Frequency mean %** **MB ML DB DL** 0 75.4**%** 72.0**%** 76.3**%** 96.6**%** 1 19.5**%** 21.2**%** 13.6**%** 3.4**%** 2 3.4% 5.1% 7.6**%** 3 0.8% 1.7% 1.7**%** 4 0.8% 0.8**%**	
Versiani et al. [22]	25 MR with middle mesial canal	Range from 0.2 to 2.4 mm in MMCs		**Brazil Turky** 1 3 (10.0%) 7 (38.9%) 2 10 (33.3%) 6 (33.3%) 3 16 (53.4) 5 (27.8%) 4 1 (3.3%) __	
Wolf et al. [61]	118		Average MR = 0.95 mm DR = 1.05 mm		In MR, wide and narrow ApC width 0.24 and 0.30 mm and 0.39 and 0.46 mm in DR
Moe et al. [60]	75			in MB 1–6 (1.55 ± 0.84) in ML 1–4 (1.59 ± 0.86) in isthmus 0–3 (0.41 ± 0.70) in MMC 0–2 (0.23 ± 0.58) **IN TOTAL 2–8 (3.78 ± 1.36)**	In MR, the largest and smallest ApC width at o.5 mm from the AF was 0.38 and 0.24, respectively
Keles & Keskin [63]	106	Average MB = 0.70 ± 0.51 ML = 0.52 ± 0.50 MMC = 1.34 ± 1.15 Whole MR = 0.85 mm			
Asijavičienė et al. [66]	60	Average MR = 1.014 mm DR = 1.089 mm All roots = 1.047 mm		**MR DR** 1 81.48% 96.67**%** 2 16.67% 3.33% 3 1.85% **___**	
Keles et al. [24]	Bifid MR (30) Non bifid MR (30)	In both bifid and non-bifid, this distance ranged from 0—2 mm in MB 0—2.5 mm in ML		**Bifid Non bifid** 2 16.7% 20**%** 3 33.3% 46.64% 4 10% 20% 5 20% 6.7% 6 16.7% 3.33% 7 3.3% 3.33%	

*MAF- Apex* Distance between major apical foramen and anatomical apex, *MAF- ApC* Distance between major and apical constriction (minor apical foramen), *MC* Mesial canals, *DC* Distal canal

As for the width of the apical constriction, it is quite plausible to see smaller and wider average dimensions in distal canals compared with mesial canals. Enclosing one canal (distal) in one root could make this difference. However, information about such dimensions would be vital in establishing the appropriate initial and master apical instrumentation sizes in every canal.

A higher number of apical foramina and the presence of many ramifications in the apical few millimetres indicate the apical canal complexity. Interestingly, in around 70% of all examined canals, no accessory apical foramina were reported [57]. Nevertheless, in the same study, there were different percentages of 1, 2, 3, and 4 apical foramina reported in mesial and distal canals. However, another study showed results that did not match those of the previous studies. The authors reported that in MB and ML canals, there were 1–6 and 1–4 foramina, respectively [60]. Table 10 shows more details regarding apical anatomy.

In conclusion, the included studies revealed that the anatomy of the mesial canals is more complicated than that of the distal canals. The difference is in the number of apical foramina per se: MB canals have more apical foramina than other canals. This can be attributed again to the wide variation in the sample sizes and differences in the populations of interest.

### Isthmuses

An isthmus is defined as ‘a narrow, ribbon-shaped communication between two root canals that contain pulp tissue’ [80]. Isthmuses within the root canal system, specifically of mandibular molars (the two-canal configuration is always associated with a high prevalence of isthmuses), might incorporate necrotic debris, tissue remnants, or organic substances that promote microbial growth, resulting in endodontic treatment failure [49]. It is probably difficult to locate the band-shaped isthmuses on 2D radiographic images prior to root canal treatment due to their buccolingual directions [62]. Notwithstanding recent advancements in the endodontic procedure, meticulous cleaning and adequate shaping of the isthmus in non-surgical approaches is still challenging. Thus, a detailed understanding of this anatomical trait of the posterior teeth is critical for successful endodontic treatment [7].

The occurrence rate of an isthmus could be related to age because it is higher in the older than in the younger age group. Additionally, it is a restricted, hardly discernible communications connecting two canals, and thus might easily be overlooked. This may exacerbate the difficulty of root-end surgical procedures of mandibular molars in elderly patients [49].

**Fig. 6:**
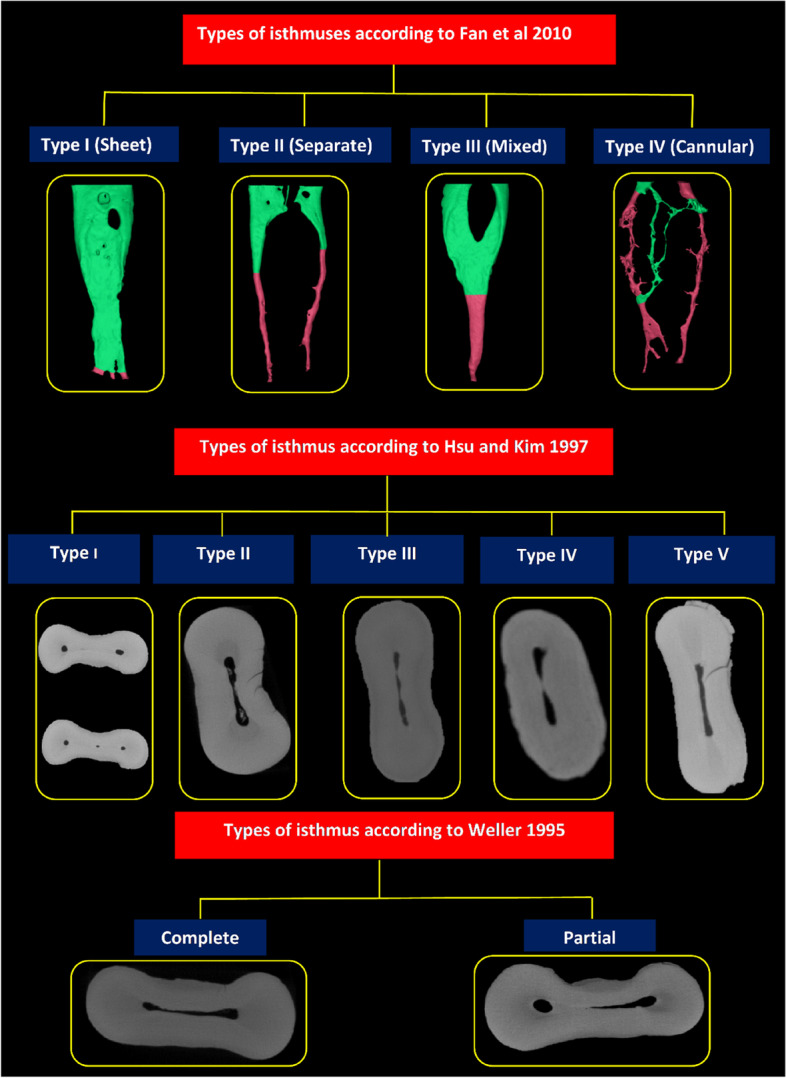
Classification systems of root canal isthmus (green colour; canal with isthmus, pink colour; canal without isthmus)

The prevalence of isthmus in the mesial root was found to be highly variable, ranging from 10% [24] to 100% [52], with a possibility of occurrence expected anywhere along the root. Variation in the level of isthmus communication among different kinds may be due to varying degrees of dentine fusion throughout tooth formation, dentine deposition, and calcification [51]. In their micro-CT study, Fan et al. [51] categorised the isthmus into four morphologies: type I (sheet connection), type II (separate), type III (mixed), and type IV (cannular connection) (Fig. 6).

According to the morphological features, Hsu and Kim [81] classified isthmuses into five categories: In type I configuration, two or three canals are present, but there is no noticeable communication. In type II, two main canals are connected with true communication. The only difference between type III and type II is the presence of three canals instead of two. In type IV, canals extend to the isthmus area, whereas type V is defined as a true communication or corridor throughout the section (Fig. 6).

Weller et al*.* [80] classified isthmuses into complete or partial on the basis of whether the narrow opening between two main root canals is continuous or incomplete through the cross section. They defined a complete isthmus as ‘one with a continuous, narrow opening between the two main root canals and a partial isthmus as ‘an incomplete communication with one or more patent openings, through the section, between the two main canals. However, the studies by Weller et al. and Hsu and Kim were limited to microscopic assessments in a 2D plane, which resulted in both specimen destruction and the loss of some of the specimen material during sectioning [51]. In this review, two studies have proved the lack of fitness in case of using the Hsu and Kim classification system, and alternatively, using the Weller system would be more useful in describing the morphology of isthmus (partial or complete) [47, 60].

Regardless of some exceptions, there has been a general consensus that isthmuses are more frequent in the middle and apical thirds of the canals [7, 47, 49, 66] and more common in mesial compared with distal canals[24, 52, 82]. Separate and mixed isthmus types are more common in mandibular first molars, while sheet connections are more common in mandibular second molars [51]. Table 11 shows a summary of isthmus results in the mandibular first molar.

**Table 11:** Summary of root canal isthmus in the mandibular first molar

**Studies**	Whole tooth/ root (N)	Isthmus classification system	Isthmus n (%)								
			**In total**	**Type I**	**Type II**	**Type III**	**Type IV**	**Type** **V**	**PI**	**CI**	**PI/CI**
Mannocci et al*.* [47]	MR (20)	Weller	17 (85) in the apical 5 mm								
Gu et al*.* [49]	MR (36)	Weller	32 (88.8) in the apical 6 mm						Grp A (85.5%) Grp B (87.5%) Grp C (94.4%)	Grp A (14.5%) Grp B (12.5%) Grp C (5.6%)	*Grp A (5.9:1) *Grp B (7.0:1) *Grp C (17.1:1**)**
Fan et al*.* [51]	MR (70)	Fan	60 (86) in the apical 5 mm	15 (23)	19 (29)	14 (21)	18 (27)				
Moe et al*.* [60]	MR (75)	Weller (In the apical 6 mm**)**	58% in the apical 6 mm						(47%)	(11%)	(5.84%)
Marceliano-Alves et al*.* [7]	MR (104)	1. Hsu & Kim 2. Fan		55(52.9) (15.4%)	8(7.7) (52.9%)	3 (2.9) (5.8%)	20(19.2) (46.2%)	18(17.3) N/A			
Asijavičienė et al*.* [66]	Whole tooth (60)	Fan	48(80)	21 (35)	21 (35)	12 (20)	10 (16.68)	N/A			
Keles et al*.* [24]	Bifid MR (30) Non bifid MR (30)	(AAE, 2020)	3(10) in non-bifid 2(6.6) in bifid roots								

*N* Number of the roots or teeth, *n (%)* Number and percentage of isthmus, *MR* Mesial roots, *PI* Partial isthmus, *CI* Complete isthmus, *Group A (20 to 39 years), *Group B (40 to 59 years), *Group C (≥ 60)

Forest plots of meta-analysis for the studies that adopted Fan's classification in this review (Fig. 4 a-d) shows higher levels of heterogeneity (I^**2**^ = 87%) among the studies, indicating inconsistent outcomes. It is well known that different populations/ethnicities, varied sample sizes, and different selection criteria could have an impact on the variability of a given anatomical entity.

### Accessory canals

According to the Glossary of the American Association of Endodontic (AAE) for Endodontic Terminology, an accessory canal ‘is a branch of the main pulp canal or chamber that communicates with the external root surface’. Thus, it is also reasonable to consider the lateral canal as a form of an accessory canal that occurs in the middle or coronal third of the root, which usually extends laterally from the main canal space. A furcation canal is ‘an accessory canal located in the furcation’ [75]. ‘An apical delta is the multiple accessory canals that branch out from the main canal at or near the root apex’. Accordingly, apical delta (or apical ramifications) has been defined by the AAE as ‘the region at or near the root apex where the main canal divides into multiple accessory canals (more than two)’ [75]. Accessory canals are common entry points for bacteria, resulting in endodontic treatment failure and the necessity for further surgical intervention.

This anatomical trait is highly prevalent in the mandibular first molar; it could reach up to 85% of these teeth [66]. Seemingly, the anatomical variability in terms of accessory canals, apical delta, and intercanal communication is higher in mesial compared to distal root canals [52, 57]. The apical third – especially in the most apical 3 mm – has a higher frequency of accessory canals than middle or coronal thirds [52, 66]. In addition, the prevalence is higher in two-rooted compared with three-rooted mandibular first molars [12].

Accessory canals could also be classified into patent, loop, and anastomosis. The patent accessory canal is ‘any branch that leaves the main canal and communicates with the external surface of the root’. The loop accessory canal leaves and rejoins the same canal (recurrent), while the anastomoses connect two different canals (intercanal branch) [24]. The patent forms are predominantly detected between the 1^st^ and 2^nd^ mm from the root apex, and their frequency is considerably greater in bifid (*n* = 53) compared with non-bifid (*n* = 30) roots. However, loop and anastomoses types are uncommon in the apical region. Table 12 illustrates a summary of accessory canals results in the mandibular first molar.

**Table 12:** Summary of accessory canals results in mandibular first molar

**Studies**	Part of focus (N)	Accessory/lateral canals			Interconnecting canals	Apical delta
		**Root third**				
		**apical**	**middle**	**coronal**		
Gu et al*.* [12]	Whole tooth (25)	MR (91.4%) DR (80%)	MR (4.3%) DR (12%)	MR (4.3%) DR (8%)		In MR 44% In DR 76%
Wolf et al*.* [57]	Whole tooth (118)	22.9% MB had (1–4) 28% ML had (1–3) 24.7% DL had (1–4) 3.4% DB had ONLY 1 AC	18.6% and 33.9% of MB and ML had at least 1(1–3) 4.2% of DL had only 1 4.2% of DL had (1–2) AC	14.3% MB had (1–3) 10.1% ML had (1–3) 4.2% DL had (1–2) 100% DB had no AC		
Moe et al*.* [60]	(MR (75)	The range of lateral canals was 0–5 in MB canal, 0–3 ML, 0–3 from the isthmuses and 0–2 in MMCs				(80%)
Marceliano-Alves et al*.* [7]	MR(104)	20.2%,	19.2%	13.5%		
Asijaviciene et al*.* [66]	Whole tooth (60)	MR (69.57%), DR (90.32%) Total = 76%	MR (15.24%), DR (9.68%) Total = 14%	MR (14.49%), DR (0%) Total = 10%		
Keles et al*.* [24]	MR with bifid apex (30)	Patent = 43 Loop = 0	Not studied	Not studied	Anastomosis (3)	
	MR with non-bifid apex (30)	Patent = 23 Loop = 1	Not studied	Not studied	Anastomosis (5)	

*AC* Accessory canals, *MR* Mesial root, *DR* Distal root, *DB* Distobuccal, *DL* Distolingual, *MB* Mesiobuccal, *ML* Mesiolingual

### Analysis of the study specifications

#### Ethical approval

**Fig. 7:**
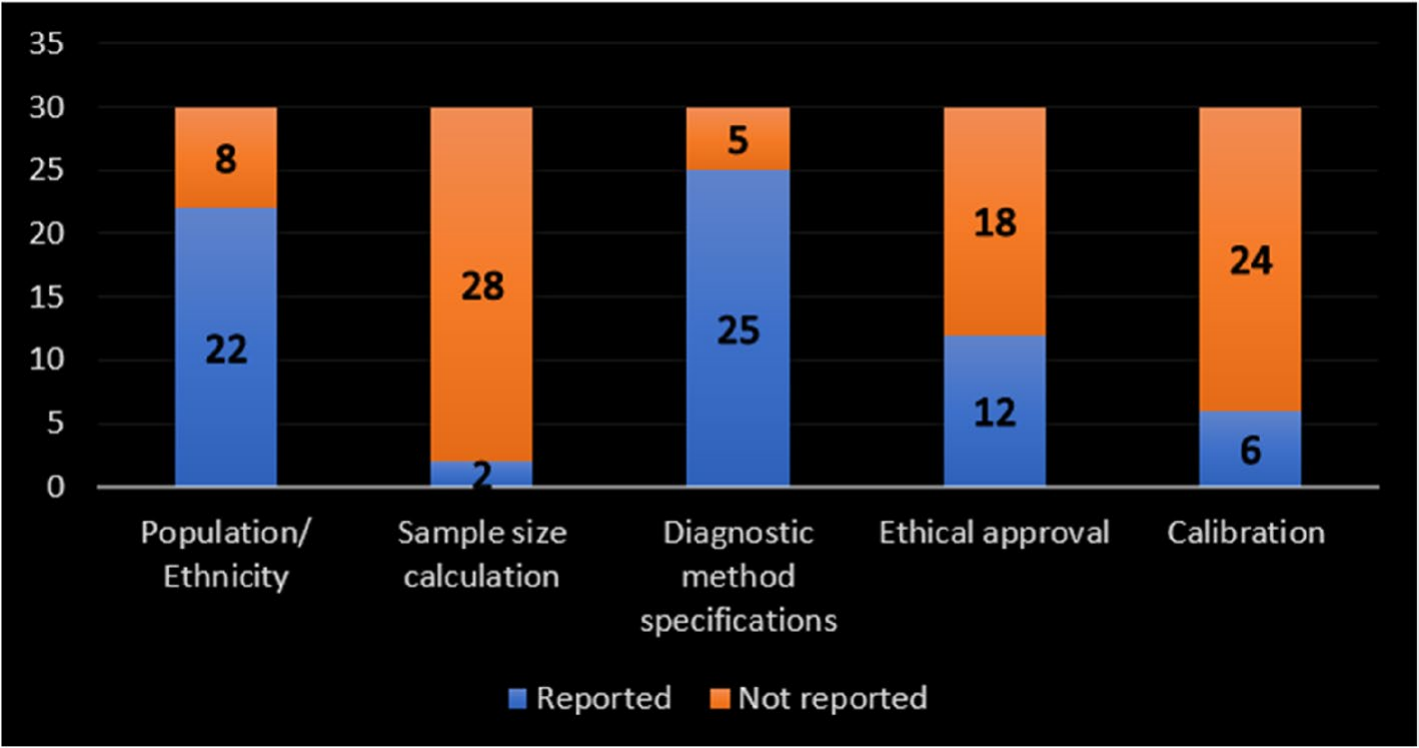
Bar chart for reporting some items

It is well known that ethical approval has no bearing on the identification of the root canal system. It may, however, improve data reliability because, along with moral and legal importance, it could provide proof of other relevant items, such as the determination of the study sample size, which is frequently stated in the application form for ethical approval [46]. Figure 7 shows the results of reporting some items in the included studies.

#### Population and/or ethnic group

**Fig. 8:**
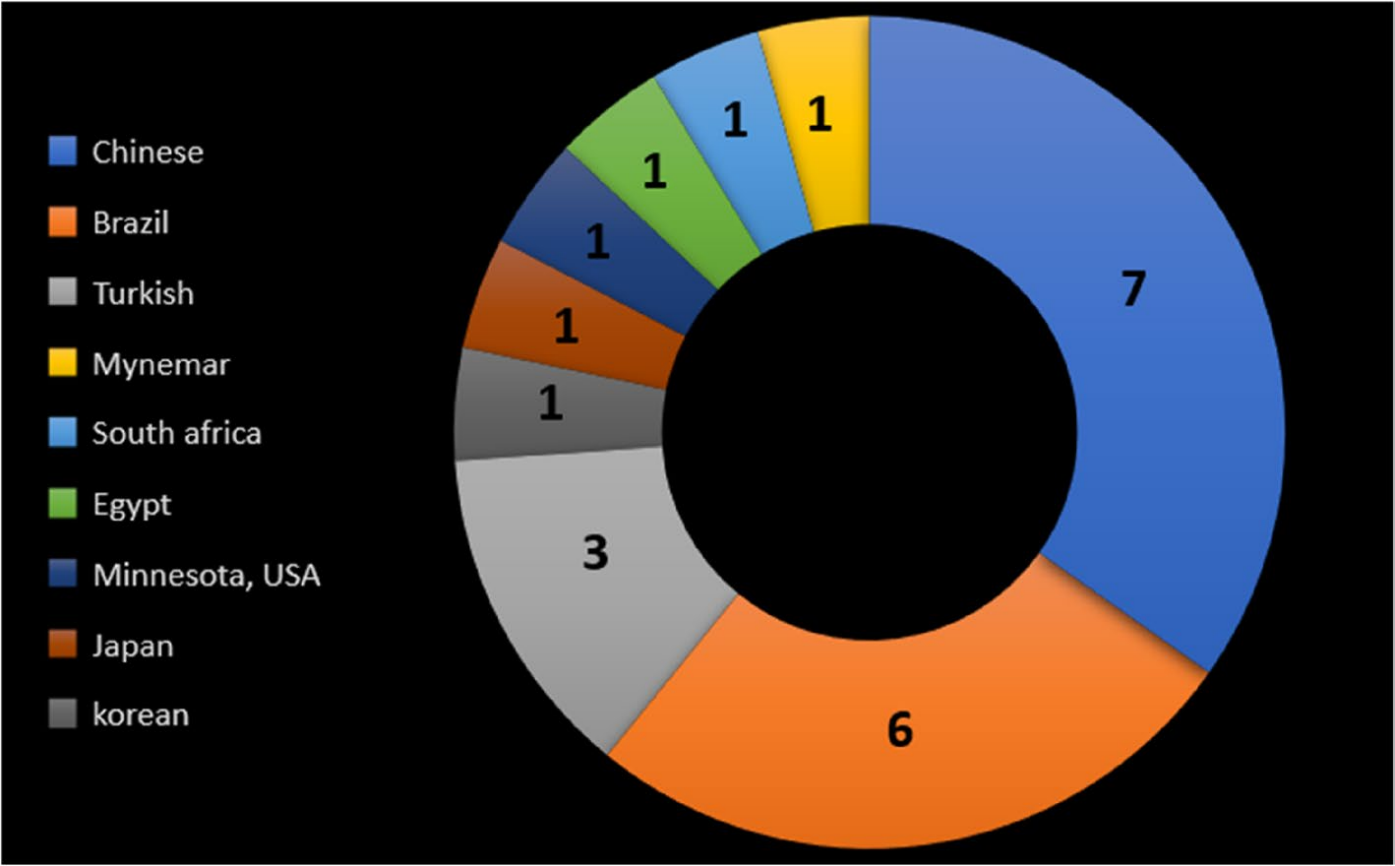
Distribution of studies on different populations/countries

According to the literature, a variety of root canal anatomical variations have been documented across ethnic groups [22]. Based on the results of this systematic review, 8 of the micro-CT root and canal anatomy studies did not report the population or ethnic group of the examined teeth (Fig. 7). The inability to identify the population and ethnicity could be due to multi-ethnic societies, making it difficult to characterise the origin of the study samples. This is particularly the case if the study had been performed using teeth that have been extracted for purposes irrelevant to the study [46]. Another possible explanation for micro-CT studies is the high cost of this imaging method, minimising the sample size to such an extent that they may not be representative of a whole population [83]. However, authors are recommended to report (in the study population section) the ethnicity/population of the area where they obtained the teeth, which might not be the same country as the authors. This would be useful for future studies and systematic reviews to have a reliable database to compare against and draw valuable findings [46]. However, Fig. 8 shows the distribution map of the population-based studies.

Clinically, understanding the population or race-related anatomical variations would assist the clinician in formulating an appropriate treatment strategy, avoiding any potential damage during root canal procedures. Otherwise, it would not be easy to relate, generalise, or apply the finding to any population unless the population of interest is clearly mentioned.

#### Type of study

Due to the fact that extracted teeth are collected from various hospitals, centres, and dental clinics, the patient information is either not documented or is poorly organised. This could explain why the vast majority of the micro-CT anatomical studies in this review had been conducted retrospectively without mentioning the age, gender, race, or reason for tooth extraction. Besides, initiating the collection of extracted teeth to fulfil the required sample size for prospectively designed anatomical investigations that aim to compare among different age groups (for example) is a challenging task. This is because such a study design would be time consuming and highly susceptible to financial and other logistic and situational constraints. Despite the useful and trustworthy information that can be gleaned from retrospective investigations, this field of study (anatomy of roots and root canals) needs additional comparative prospective studies that take into account diverse populations and ethnicities, age groups, gender, and tooth sides. This will have a significant impact on expanding knowledge, particularly regarding this challenging tooth type, paving the way and providing a strong basis not only for clinicians but also for future experimental or review analyses to derive more meaningful conclusions.

#### Sample size and its calculation method

Sample size calculation is necessary when planning research. Estimating a sufficient sample size for a given study is critical for adopting and generalising the results by drawing a valid conclusion [84]. Sample size calculation is affected by the type of study (micro-CT or other), population of interest, research question, cost, objective (qualitative and/or quantitative), and availability of resources. However, reducing the sample size to a reliable minimum may be attributed to the available time, convenience, and higher cost (as in the case of micro-CT studies) and resources [46].

The results of this systematic review show that the vast majority of the studies did not mention the sample size calculation method (Fig. 7), which could be considered a weakness. Besides, the included studies had a wide sample size range, from 19 [65] to 140 [7]. The anticipated repercussions of using a small sample size are potentially misleading findings and a wide range of variations, leading to misinterpretations or discrepancies. As long as reliable, accurate conclusions can be achieved by using fewer specimens, using an overestimated sample size would not be recommended [84].

There are several expenses associated with micro-CT studies, such as training, scanning of teeth for long hours using specific scanning parameters (such as number of projections and voxel size), registration fees for dedicated software programs, as well as maintenance of the micro-CT machine. Additionally, the study per se might be restricted to a limited time and budget [46]. All of the issues listed above could make it hard to include more samples in a micro-CT study.

The sample size calculation, whether it was not conducted or conducted but not reported, is a weak point in most micro-CT studies. The sample size and its calculation approach must be provided to prove that the findings represent a specific population of a given tooth type.

#### Diagnostic method specifications

Based on the included studies, micro-CT has been used efficiently to provide adequate quantitative and/or qualitative analysis and, thus, to generate valuable, reliable, and more detailed information about the target entity. Micro-CT has accurately evaluated a wide range of objectives (root morphology, canal configuration, isthmus, accessory canal, and pulp chamber floor morphology (Table 3). The scanning procedure consists of many parameters (specifications), such as Kv, µA, power, voxel size, type of filter, and others. Experimenting with and adjusting these parameters would invariably predict the quality of the generated data. So, the best way to optimise and choose the right parameter values for a given research sample and objective would be to do a pilot test on one or a few samples.

It is noteworthy that the isotropic resolution in this review ranges from 9.9 to 50 µm. This value has decreased over time, indicating that the resolution of micro-CT scanners has improved. The finer the field or the anatomical structure of interest, the smaller the voxel size that is used. A voxel size of around 20 µm has been used efficiently to study fine details such as the number of accessory foramina; connecting and accessory canals; the distance between the major apical foramen and the root apex; and the diameter, number, and shape of the apical constriction [57]. The smallest voxel size reported in this review is in the study by Versiani et al*.* [56]. They quantitatively and qualitatively assessed the MMCs using a voxel size of 9.9 µm [22]. Although the vast majority of the micro-CT studies in this review reported these specifications, others failed to report them, and this would undoubtedly be considered a deficiency. So, from our point of view, the diagnostic method parameters (specifications) need to be reported and described clearly in the ‘Materials and Methods’ section of anatomical studies. However, Fig. 7 shows the results of reporting some items in the included studies.

#### Calibration

In light of the fact that each evaluation method has limitations and may necessitate certain forms of training, calibration is crucial for achieving reproducible and reliable outcomes. Clinical and experimental observations must be assessed accurately by investigators that have been preferably calibrated over time so that the findings can be interpreted confidently. This is especially important for inspecting root canal configurations, canal orifice morphology, changes in 2D or 3D radiographs, and data from 3D reconstructions [22, 46]. Therefore, calibrating the data is a vital step in the process of validating the results – by minimising the potential bias and decreasing error percentage during a qualitative or quantitative evaluation of any anatomical entity. The majority of studies (24 out of 30) in this review did not report the calibration procedure, whereas in 6 studies, the authors had calibrated their data (Fig. 7). Calibration is needed to reduce the chance of human error as well as for devices and/or software that can be used to get certain measurements. To do so, one, two, or more trained observers could be involved to assess certain parameters and average the recorded values. Notably, intra-observer agreement/reliability (same observer repeats the same assessment on different occasions) is preferable with studies that involve quantitative analysis [12, 85]. On the other hand, inter-observer agreement/reliability (two or more observers making the same assessment at a specific time point or period) is preferable for those that have qualitative or mixed analysis [4, 49]. In micro-CT-based anatomy investigations involving qualitative objectives with a high potential for subjectivity, the evaluation procedure would be strongly dependent on the convenience and consistency of the observer. Consequently, the more the potential subjectivity, the greater the number of observers necessary for data viewing and interpretation. For example, evaluation of the apical constriction (minor apical foramen) form on 3D-reconstructed views (which can exhibit different forms when viewed from different angles) requires two or more trained observers (up to five) [86, 87]. However, greater subjectivity can be attributed to the lack of standardisation, the absence of explicit assessment standards, and the absence of obvious demarcations between different anatomies. Indeed, Cohen’s kappa agreement test is a valuable tool for performing this task, and substantial or almost-perfect agreement is extremely desirable to reinforce and validate the research outcome [85]. Because root and canal anatomy is the topic of concern, it is essential that the researcher always be calibrated for evaluation methodologies and categorisation of anatomical entities (like canal configuration, isthmus, accessory canals, etc.).

### Clinical implications

**Fig. 9:**
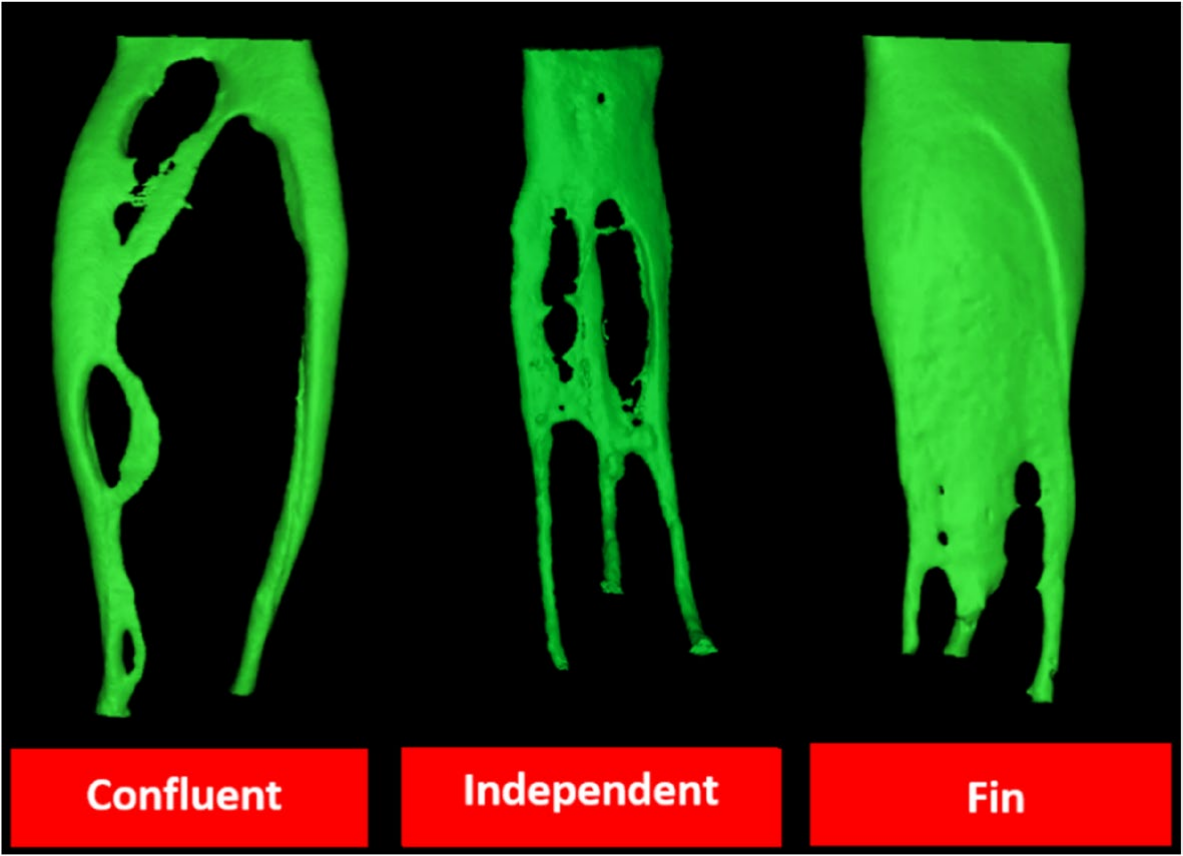
Morphologies of middle mesial canals (MMCs). Confluency and tortuous path of MMC increases its cleaning and disinfecting difficulties

The authors advocate that understanding of the normal and complicated root and canal anatomies has a direct impact on the clinical scenario. Given that the success or failure of root canal treatment is closely related to how to correlate the challenging anatomy with treatment protocols, several clinical implementations have been derived from the existing literature.

i. The diversity of root canal anatomy has an impact on the instrumentation, irrigation, and obturation techniques. For example, clinicians should follow the recommended guidelines for detecting MMCs to prevent post-treatment infections. Vertucci type II and IV have been the most predominantly reported configurations in mandibular first molars. Nevertheless, other more complex configurations could also be expected, especially in the mesial roots. This greater complexity that has been reported in the mesial compared with the distal root canals might pose extra challenges, affecting the long-term prognosis of the root canal treatment [4].
ii. Accessing an additional canal has been facilitated greatly by a thorough inspection of the grooves connecting the two mesial canal orifices. Nevertheless, the length and depth of this groove must be taken into account because of the presence of thin root dentine at the furcal side of MMCs, which raises the risk of root perforation. An independent MMC may be an issue in clinical practice if its orifice is not located even after a troughing process. However, using new files with minimum tapers to navigate such narrow MMCs might be useful in debriding it with less potential damage to both canal and instrument. Fin and independent configurations could be rather simple to prepare and disinfect. In the case of the confluent configuration (Figure 9), however, complete debridement of the canal system is more challenging [56]. To minimise the potential treatment failure and to prevent the necessity for further retreatment or surgical intervention, the endodontist must focus their efforts on delivering and activating irrigants as efficiently as possible to accomplish adequate disinfection.
iii. When a tooth has a long-oval or flattened canal, endodontic instruments would inevitably enlarge the mesiodistal aspect (danger zone) more than the buccolingual aspect of that canal. By introducing an endodontic file into a canal with such dimensions, the already thin furcal aspect of the root may be further reduced, while the buccal and lingual walls could remain uninstrumented. Additionally, not all recesses of irregular or oval-shaped root canals could be accessible in the circular preparation generated by the rotating instrument [52]. Therefore, care must be taken during canal instrumentation or post preparation [55], and the efforts must be focused on performing a highly effective irrigation and agitation protocol. Because the roundness values in distal canals decrease in the coronal direction (flattened canal), it is advisable to prepare the single distal canal as two separated canals [88].
iv. Dental practitioners must be aware of the length and curvature of roots because highly curved roots increase the likelihood of instrument separation or ledge formation. Moreover, the operator could expect the presence of greater anatomical complexity in the root canal system of mandibular first molars with long roots [11]. Therefore, prior to initiation of root canal treatment, a thorough inspection of diagnostic radiographs is indispensable to achieve successful treatment outcomes.
v. Understanding the root apex morphology is critical for endodontists to build an appropriate treatment plan. The preparation of root canals should extend to the apical constriction. Hence, as evidenced by experimental research and biological considerations, exceeding the minor apical foramen (apical constriction) during cleaning, shaping, or obturation must be avoided. Moreover, care must be taken during the working length estimation (WLE) and instrumentation of MMCs as they have been reported to be shorter and more deviated from the apices compared to MB and ML canals [22]. As a result, clinicians are recommended to carefully use the electronic apex locator to determine the working length. The presence of apical accessory foramina in the mesial root indicates higher apical complexity and a higher number of apical ramifications. Therefore, upon endodontic surgery, particularly on mesial roots, clinicians are recommended to remove most of these apical ramifications (by removing the most apical 3 mm of the root) to achieve a better postoperative outcome.
vi. A higher occurrence rate of the isthmus has been reported in the apical third of mesial roots in mandibular first molars. To guarantee a good apical seal following apical root resection, the root-end preparation should reach at least 3 mm into the canals. Literature observations have indicated that the 3-mm-deep root-end preparations could be executed frequently in regions (apical 4–6 mm) occupied by isthmuses. So, it should be standard clinical practice to include the isthmus in root-end preparation and root-end filling to make sure that the root canal is cleaned and sealed properly [49]. A large number of microorganisms and remaining pulp tissues can be found in larger isthmuses, which may contribute to treatment failure. Cleaning and preparing a flattened or long oval-shaped root, particularly those containing isthmuses, is challenging [49].

### Future directions

Micro-CT has high resolution and could be more useful and efficient in revealing fine and intricate details such as isthmuses and lateral canals. However, as mentioned before, the disadvantages of this imaging technique are that it is time consuming, costly, and its use is limited to extracted teeth. Due to these limitations, the sample size for micro-CT investigations is frequently kept to a reasonable minimum; consequently, the anatomical findings among studies can vary significantly. Thus, additional research on the 3D anatomy of the teeth would enrich the existing and published data. To conduct micro-CT studies focusing on root and canal anatomy with high level of accuracy, quality, and reliability, the Preferred Reporting Items for Study Designs in Endodontology (PRIDE) guidelines have been introduced [89]. Moreover, in an effort to minimise inconsistencies in reporting the items in any anatomy study, it is recommended to adhere to the Preferred Reporting Items for Root and Canal Anatomy in the Human Dentition (PROUD 2020) checklist that have been established for the preferred reporting items relevant to anatomical investigations [46].

**Fig. 10:**
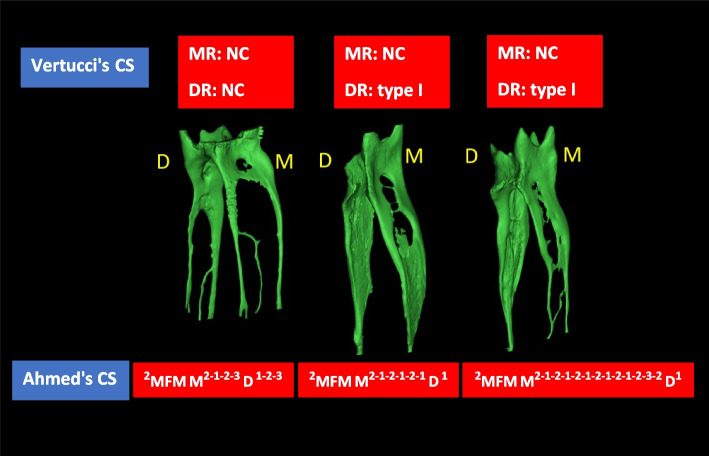
Ahmed's vs Vertucci classification systems. Superscript 2; two rooted molars; MFM: mandibular first molar; M: mesial; D: distal; superscript 1-2-3 = superscript O-C-F: (**O**) number of orifices, (**C**) canal configuration, and (**F**) number of main foramina: MR: mesial root; DR: distal root; CS: classification system; NC: non classifiable

**Fig. 11:**
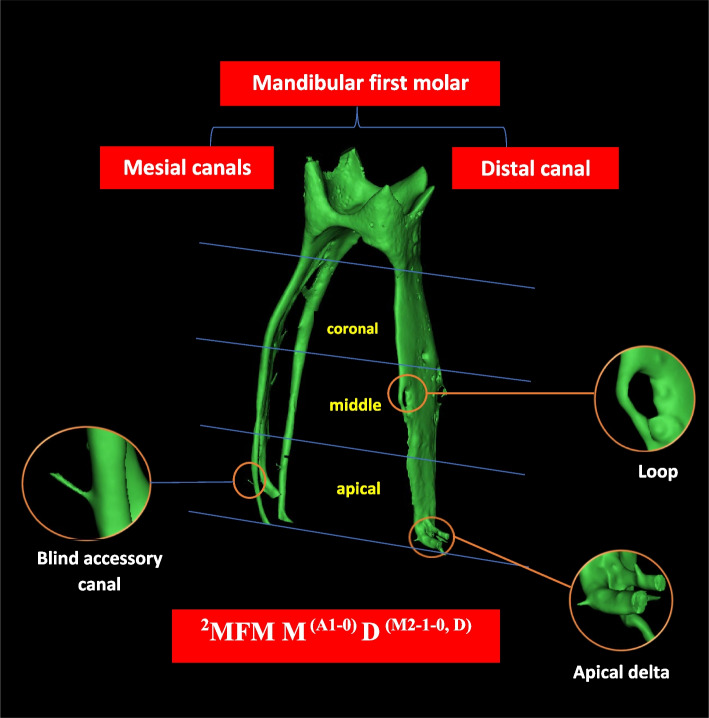
New coding system for evaluating accessory canals. Superscript 2: two rooted tooth; MFM: mandibular first molar; M: mesial; superscript A1: presence of one patent accessory canal at the apical third; D: distal; superscript M2-1-0: presence of loop at the middle third of the canal; superscript D: apical delta

Based on the authors’ understanding of the current literature, this section provides general future directions to assist investigators who are interested in evaluating the anatomy of the mandibular first molar using micro-CT imaging technology. A number of critical points are addressed to facilitate future enhancements, using new classification systems, including more objectives, and even comparing a given feature (or features) in different populations, expanding and establishing reliable and accurate knowledge about this highly challenging tooth type.

i. The recently proposed coding classification system [76] to describe the morphology of the root and canal system (Fig. 10) has been proven to be more accurate and practical, compared with Vertucci's system [35, 90]. Using this system, especially in micro-CT-based studies, could enable researchers to accurately describe and to present all the possible configurations, and no configuration would be left as non-classifiable. This coding system can also be used for describing and evaluating the presence, location, number, and configuration of accessory canals [75] (Fig. 11). Moreover, the presence of root anomalies can also be described in conjugation with this coding system [91].
ii. New valid, reproducible, clinically relevant, and evidence-based criteria should be proposed to define some confusing and challenging anatomies, such as differentiation between main and accessory canals beyond the area of apical canal bifurcation and determining the level of canals' orifices.
iii. The apical constriction (minor apical foramen) is not a plane or a point, it is a 3D object, and it is not confirmed that it is perpendicular on the long axis of the canal. For instance, it had been found that 66.7% of the apical constrictions had asymmetrical appearance (i.e., AC were not perpendicular on the canal axis) [87]. Subsequently, a detailed micro-CT-based 3D assessment of the apical morphology is warranted to establish a new approach for precising the localisation and allowing more accurate qualitative and/or quantitative evaluation of this anatomical trait.
iv. Given that the values obtained from the quantitative 2D & 3D measurements could have direct (e. g., linear distances from the major and/or minor apical foramen to the root apex and roundness and form factor parameters) or indirect (such as Surface Model Index [SMI]) clinical implementations as discussed in a recent review [83]. Researchers and clinicians are highly encouraged to collaborate and conduct laboratory studies with clinical translation (such as methods of detection and preparation of different canal configurations), transferring such important relevant information into a clinical setting.

### Limitations

This systematic literature review (SLR) has limitations since it only included micro-CT studies that involved only extracted teeth in which the gender and age were unknown. Additionally, the search process was restricted to English-language articles. Nonetheless, evidence suggests that searching for non-English papers rarely changes the conclusion of systematic reviews [92].

## Conclusions

There is a considerable heterogeneity in the methodological procedures used to study root canal anatomy in micro-CT studies. However, all involved studies showed that the root canal anatomy of the permanent mandibular first molar is highly variable. Such anatomical complexity requires awareness even for the well-trained clinicians, attention with diagnostic approaches and more practical skills for getting a successful root canal treatment outcome. The results of this systematic review showed that the variety in population, methodology used, inclusion and exclusion criteria, and sample size could attribute to the anatomical variability of this tooth type. More collaboration between researchers and clinicians is required in order to translate and apply the information derived from research (theoretical frame) to the clinical scenario and conduct more clinical studies to enrich and expand knowledge about the anatomical variability of this tooth type.

## Data Availability

The datasets used and/or analysed during the current study are available from the corresponding author on reasonable request.

## Abbreviations

2D: Two-Dimensional
3D: Three Dimensional
SEM: Scanning Electron Microscope
CBCT: Cone-Beam Computed Tomography
Micro-CT: Micro-Computed Tomography
PRISMA: Preferred Reporting Items for Systematic Reviews and Meta-Analysis
MMCs: Middle Mesial Canals
AQUA tool: Anatomical Quality Assessment tool
µA: Milliamperage
Kv: Kilo voltage
CEJ: Cemento-Enamel Junction
MR: Mesial Root
DR: Distal Root
MB: Mesiobuccal
ML: Mesiolingual
DB: Distobuccal
DL: Distolingual
CS: Classification System
NC: Non-Classifiable
IOD: Inter-Orifice Distance
DZ: Danger Zone
MAF: Major Apical Foramen
ApC: Apical Constriction
